# Infrared-reflective ultrathin-metal-film-based transparent electrode with ultralow optical loss for high efficiency in solar cells

**DOI:** 10.1038/s41598-023-50988-3

**Published:** 2024-01-04

**Authors:** George Perrakis, Anna C. Tasolamprou, George Kakavelakis, Konstantinos Petridis, Michael Graetzel, George Kenanakis, Stelios Tzortzakis, Maria Kafesaki

**Affiliations:** 1 Institute of Electronic Structure and Laser (IESL), Foundation for Research and Technology - Hellas (FORTH), 70013 Heraklion, Crete Greece; 2 Department of Physics, National and Kapodistrian University of Athens, 15784 Athens, Greece; 3 Department of Electronic Engineering, Hellenic Mediterranean University, Romanou 3, Chalepa, 73100 Chania, Crete Greece; 4 Laboratory of Photonics and Interfaces, Institute of Chemical Sciences and Engineering, Ecole Polytechnique Fédérale de Lausanne, 1015 Lausanne, Switzerland; 5 Department of Materials Science and Technology, University of Crete, 70013 Heraklion, Crete Greece; 6 Texas A&M University at Qatar, 23874 Doha, Qatar

**Keywords:** Solar energy and photovoltaic technology, Photonic crystals

## Abstract

In this work we study in-depth the antireflection and filtering properties of ultrathin-metal-film-based transparent electrodes (MTEs) integrated in thin-film solar cells. Based on numerical optimization of the MTE design and the experimental characterization of thin-film perovskite solar cell (PSC) samples, we show that reflection in the visible spectrum can be strongly suppressed, in contrast to common belief (due to the compact metal layer). The optical loss of the optimized electrode (~ 2.9%), composed of a low-resistivity metal and an insulator, is significantly lower than that of a conventional transparent conductive oxide (TCO ~ 6.3%), thanks to the very high transmission of visible light within the cell (> 91%) and low thickness (< 70 nm), whereas the reflection of infrared light (~ 70%) improves by > 370%. To assess the application potentials, integrated current density > 25 mA/cm^2^, power conversion efficiency > 20%, combined with vastly reduced device heat load by 177.1 W/m^2^ was achieved in state-of-the-art PSCs. Our study aims to set the basis for a novel interpretation of composite electrodes/structures, such as TCO–metal–TCO, dielectric–metal–dielectric or insulator–metal–insulator, and hyperbolic metamaterials, in high-efficiency optoelectronic devices, such as solar cells, semi-transparent, and concentrated systems, and other electro-optical components including smart windows, light-emitting diodes, and displays.

## Introduction

Visibly transparent conductors or composite structures such as TCO-metal-TCO^1–3^ (TCO: transparent conductive oxide), dielectric-metal-dielectric^4–8^ (DMD) or insulator–metal–insulator (IMI), and hyperbolic metamaterials (HMMs)^8–10^ have recently emerged as highly-promising and feasible structures for efficient heat-insulation/filtering^6,8–11^ (i.e., via reflecting infrared light), radiative cooling^6,9–11^ (i.e., via emitting thermal radiation in the atmospheric transmission window), and electrical conduction^1–5,7,12^ (mainly due to the metal thin film of high conductivity) in a wide range of optoelectronic devices and components including solar cells^2–4,11,13^, smart windows^9–11^, light-emitting diodes^11,14^, and displays^11,14^.

However, their trade-off relationship between the electrical sheet resistance (*R*_s_) and the transmittance (*T*) has so far impeded their application in high-efficiency optoelectronic devices such as high-efficiency solar cells^2–4,11,13^. Specifically, ultrathin metal films (< 15 nm) present very low *R*_s_ which can be tuned by varying the metal thickness. However, their transmittance in the visible spectrum (~ 400–800 nm) is low, mainly due to reflection from the compact metal layer, unless properly-designed antireflection (AR) metal undercoat and overcoat layers are applied^5,12^. More drawbacks such as the large surface roughness and high optical scattering, adversely affect the *T*–*R*_s_ trade-off, leading to uncompetitive *T*–*R*_s_ values^8^.

The in-depth study on the AR properties of composite structures on glass and polymer substrates as well as recent advances in material processing and adhesion^5,12^, have led to a plethora of promising composite structures with ultra-high transparency in the visible spectrum (> 90%)^5,8,12^, combined with very low *R*_s_ (< 15 Ω/sq)^1,3–5,7,12,13^ and efficient reflection of infrared (IR) radiation (> 50%)^6,8–11^. Proposed structures are composed of a variety of metals and encapsulation materials depending on the application, and may involve Ag^5,12^, Au^13^, Cu^11^ and TiO_2_^8^, SnO_2_^11^, ZnO^5^, AZO^7,12^ or ultra-thin (< 50 nm) ITO^2^, GZO^3^, and FTO^1^, placed on top of various substrates, such as glass wafers^5,8,10,12^, PEN^4^ or PET foils^5,7,12^. Despite the high *T* and low *R*_s_ reported, the integration of such structures in high-efficiency optoelectronic devices like high-efficiency solar cells (e.g., perovskite-, polymer-, or silicon-based solar cells), still leads to low photocurrent (*J*_ph_ < 20 mA/cm^2^)^2–4,11^ and therefore low solar-to-electrical power conversion efficiencies (PCE < 18%)^2–4,11,13^.

The enhanced transmittance of ultrathin metal films relies on the destructive interference of the reflected waves where the reflectivity dip is controlled by the overcoat and undercoat layer thicknesses^5,12^. In multilayer systems such as solar cells, the material, thickness, and number of thin-film layers below the composite electrode (i.e., hole- and electron-transporting layers, passivation layers, or semiconductor materials) naturally affect light interference. However, most thorough studies so far examine the design and spectral response of composite structures on glass or polymer substrates^5,8,10,12^. Additionally, solar cell studies consider a low number of metal encapsulation layers (only undercoat and overcoat). This is expected to lead to a narrower transmission band width along the visible spectrum, which significantly affects *J*_ph_ in solar cells.

Here, we study in-depth the antireflection and filtering properties of ultrathin-metal-film-based multilayer transparent electrodes (MTEs) integrated in thin-film solar cells, and show that, through proper optimized design of the total (electrode-cell) system, reflection in the visible spectrum can be strongly suppressed. The optical loss of the optimized electrode (~ 2.9%), composed of a low-resistivity metal and an insulator, is significantly lower than that of a conventional TCO (~ 6.3%) and comparable to a single-layer Graphene (~ 2.3%)^12^, thanks to the very high transmission of visible light within the cell (> 91%) and low thickness (< 70 nm), whereas the reflection of infrared solar light (~ 70%) improves by > 370%. Such an optical performance is, to our knowledge, one of the highest reported so far in solar cells with front-contact electrodes of compact metal layer^2–4,11,13^. Additionally, its potential in realistic solar cells is demonstrated by showing that it can serve as an ultra-thin transparent front contact and a highly-efficient IR filter in state-of-the-art perovskite solar cells (PSCs), with *J*_ph_ > 25 mA/cm^2^, PCE > 20%, and vastly reduced device heat load by 177.1 W/m^2^. While the proposed methodology is performed on various designs and configurations of multilayer PSCs, the proposed strategy is generic to all solar cells with optimal band-gap (~ 1.4–1.5 eV), aiming to set the basis for a novel interpretation of composite electrodes/structures in high-efficiency optoelectronic devices, such as solar cells, semi-transparent, and concentrated systems, and other electro-optical components including smart windows, light-emitting diodes, and displays.

The manuscript is organized as follows: Initially, we present MTEs’ and PSCs’ structure and discuss the considerations and modeling to optimize MTEs’ design. Then, we demonstrate the optical, electrical, and thermal performance of optimized MTEs (i.e., transparency, IR filtering, and radiative cooling effect on PSCs’ PCE and operating temperature) and elucidate the requirements for high performance beyond the-state-of-the-art. Finally, we provide a detailed description of the fabrication, characterization, and modeling methods employed for structures’ optimization.

## Results

### Structure of MTE electrode and modeling.

**Figure 1:**
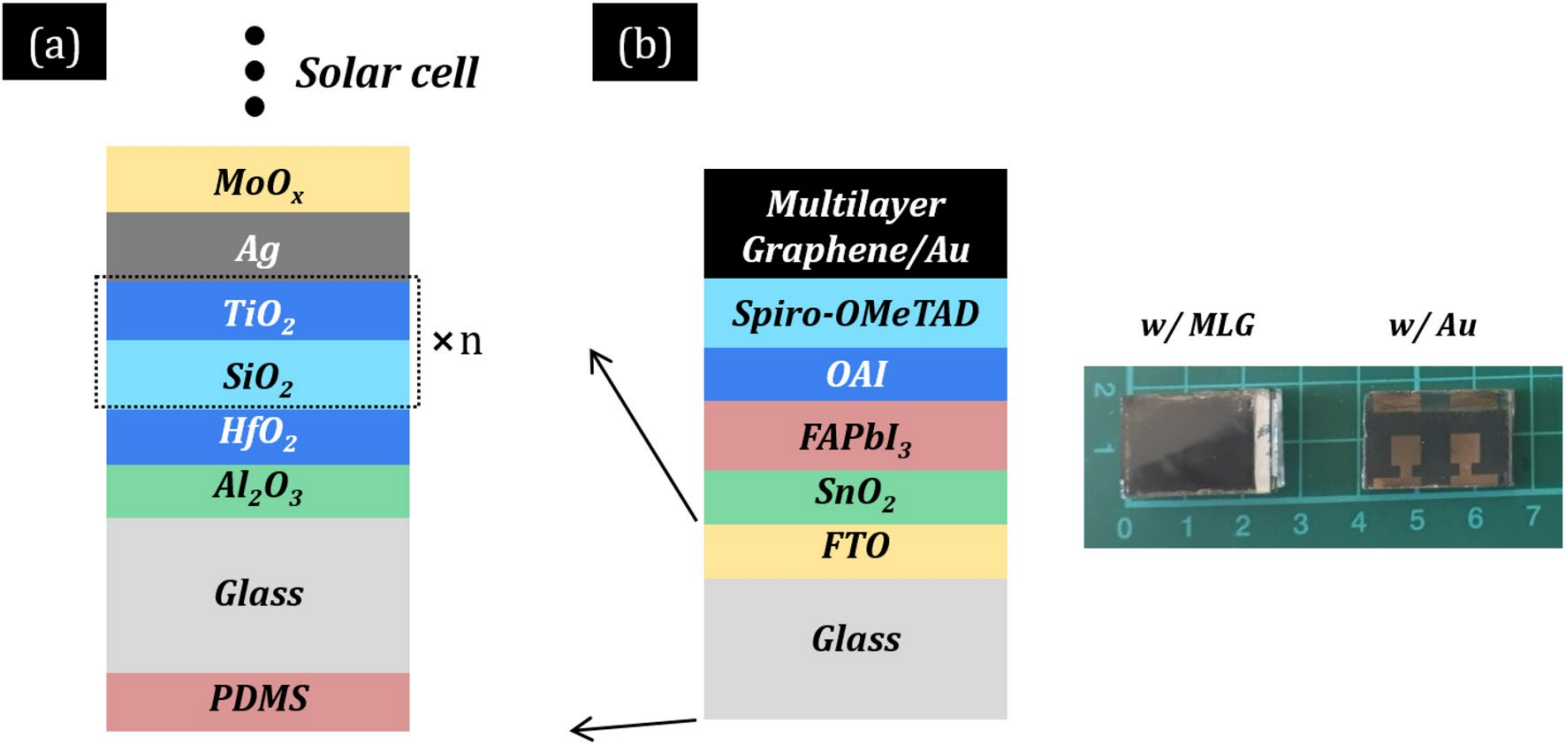
(**a**) The designed ultrathin-metal-film-based multilayer transparent electrode (MTE) with PDMS/Glass/Al_2_O_3_/HfO_2_/alternating SiO_2_-TiO_2_/Ag/MoO_x_ structure for reflection of the ultraviolet (UV), near-infrared (NIR), and short-wave-infrared (SWIR) radiation, enhanced transparency in the visible spectrum (VIS), maximum thermal radiation in mid-infrared (MIR), and low sheet resistance (*R*_s_). (**b**) Geometry of the thin-film PSCs investigated here (left). The role of the different layers of the solar cells is discussed in the main text. Photo of the back side of the two measured solar cells (right). Cell “w/MLG”: a perovskite solar cell with a multilayer Graphene back contact. Cell “w/Au”: a perovskite solar cell with Au back contacts.

The structure of the proposed ultrathin-metal-film-based multilayer transparent electrode (MTE) deposited on a soda-lime glass substrate is shown in Fig. 1a. Other transparent substrates including other glasses and polymers can be used. The proposed design was selected to provide (i) high photocurrent, i.e., through high solar transmission in the visible spectrum (VIS), (ii) reduced heat source, i.e., via reflecting harmful ultraviolet (UV), near- (NIR), and short-wave-infrared (SWIR) sun radiation, and (iii) enhanced radiative cooling, i.e., through enhanced emissivity in mid-infrared (MIR) within the atmospheric transmission window^15,16^. High photocurrent and lower operating temperature (i.e., due to reduced device heat load and enhanced cooling) are expected to increase solar cells’ PCE^15–17^.

Among the metals, we chose Ag as it has the highest electrical conductivity and lowest absorption loss. Ag overcoat is in direct contact with the solar cell. We used molybdenum oxide (MoO_x_) as an overcoat layer because of its relatively high refractive index (~ 1.9) and the fact that its low conductivity ensures electrical contact between the Ag film and the solar cell, which is essential for the functionality of the device. Depending on the architecture and processing conditions, other materials may also be utilized such as TiO_2_^8^, SnO_2_^11^, ZnO^5^, AZO^7,12^, or ultra-thin (< 50 nm) ITO^2^, GZO^3^, or FTO^1^. Ag undercoat consists of one or more dielectric layers in our study to provide a broader transmission band width along the visible spectrum, which is crucial for achieving high photocurrent, and high-performance filtering function. Specifically, it consists of alternating layers of titanium dioxide (TiO_2_), silicon dioxide (SiO_2_), aluminum oxide (Al_2_O_3_), and hafnium dioxide (HfO_2_) of varying thicknesses, which are all deposited on the glass substrate (see Fig. 1a). These layers assist in (i) suppressing reflection from the metal and obtaining broadband transparency in the beneficial wavelength range where photons convert to photocurrent (~ 0.385–*λ*_g_ μm, where *λ*_g_ is perovskite’s band gap wavelength), and (ii) optimizing reflection of the UV (~ 0.3–0.385) and IR light (~ *λ*_g_–4 μm) that only heats the solar cell, in a manner similar to that achievable using periodic 1D photonic crystals. TiO_2_ and HfO_2_ are the high-index materials^16^, while SiO_2_ and Al_2_O_3_ are optically transparent and are the low-index and antireflection layers, respectively. To enhance radiative cooling, we coated the glass substrate (front side) with a common inexpensive polydimethylsiloxane (PDMS) film that maximizes thermal radiation in the atmospheric transmission window. As we show next, to optimize the MTE design, we employed an optical-electrical-thermal modeling and performed an optimization procedure (see also Methods section for a detailed description). To ensure validity of our simulations, we fabricated two state-of-the-art PSC samples and characterized their absorptivity/emissivity spectra in the visible and infrared wavelength range (see also Supplementary Information for more details).

Due to light interference in thin-film solar cells, we optimize the thickness of each layer of the MTE and the cell assuming that the MTE is integrated in the solar cell. The optimization of the total (electrode-cell) system is implemented by combining a global evolutionary algorithm (“Genetic” algorithm) and a local optimization method (“Nelder Mead Simplex”)^16^ over an objective function, cells’ PCE calculated according to the theoretical model discussed below (see also Methods section for a detailed description). We consider PCE a more accurate metric to evaluate the influence of MTE on solar cells than *J*_ph_, since besides *J*_ph_, light interference in the thin film stack strongly affects parasitic absorption in other layers than the photoactive material where carriers are generated, hence heat generation and operating temperature (*T*_c_) which in turn affect PCE. The independent variables (optimized according to the objective function) are the thicknesses of each layer of the MTE and the cell, where in our case the materials are predefined.

For the MTE, we examined three Ag thicknesses of 8, 10, and 12 nm that provide low *R*_s_ (< 15 Ω/sq). The overcoat was kept thin with thickness varying in the range 10–60 nm to ensure low series resistance. Since the undercoat is not in direct contact with the solar cell, film’s thicknesses varied in the range 10–200 nm. For the cell, we consider high-efficiency, thin-film solar cells, namely perovskite solar cells (PSCs), with optimal band-gap (~ 1.4–1.5 eV) and PCE > 20%. We consider two types of state-of-the-art PSCs with typical *n*-*i*-*p* stack layout (upside down fabrication on top of a glass substrate—see Fig. 1b): cell denoted by “w/Au” is a PSC with Au back contacts for enhanced efficiency, and cell denoted by “w/MLG” is a PSC with a multilayer Graphene back contact for enhanced stability and low cost. Specifically, cell configuration w/Au exhibits record efficiencies, even beyond 25% for smaller cells^18,19^, but in expense to the increased cost and reduced operational stability^20,21^. Cell configurations w/MLG exhibit lower efficiencies (~ 20%)^21,22^, mainly due to open-circuit voltage (*V*_OC_) loss as a result of hole trapping due to poor contact (pinholes and gaps) at the hole-transporting layer (HTL)-MLG interface (see Fig. 1b)^22^, but high device operational stability and lower cost due to high-throughput fabrication, e.g., by utilizing printing techniques and low-cost materials^20,21,23^. The rest of the structure is as follows: TEC 8 (FTO—500 nm)-covered glass substrate (FTO is replaced by MTE in our case)/tin oxide (SnO_2_—10 to 30 nm)/perovskite (FAPbI_3_—350 to 800 nm)/Octylamonium iodide (OAI—5 to 10 nm)/Spiro-OMeTAD (150–200 nm)/multilayer Graphene (10 μm) or Au (80 nm)/ethylene vinyl acetate (EVA—0.47 mm)/polyvinyl fluoride (TEDLAR—0.5 mm), where the numbers indicate the thickness of each layer, and the ranges indicate the boundary values of the independent variables corresponding to typical thicknesses in experiments. For both solar cells, low-band gap formamidinium lead iodide perovskite (FAPbI_3_) is the photoactive layer for enhanced efficiency and thermal stability^18,19^. SnO_2_ and Spiro-OMeTAD are the electron- (ETL) and hole-transporting layers (HTL), respectively, OAI is the passivation layer, and commercial fluorine doped tin oxide (FTO) is used as the reference transparent electrode replaced by MTE in our case.

To calculate PCE as a function of *T*_c_, we employed an analytical, generic optical-electrical-thermal model. Optically, we calculated PSCs’ absorptivity by employing the transfer matrix method and used it as a heat input in the electro-thermal simulation. We then set up a coupled electro-thermal simulator solving the steady-state energy balance for solar cells, with which we simulate the PCE as a function of *T*_c_, assuming varying ambient temperature, humidity, and wind speed to mimic typical outdoor conditions (see Methods section for a detailed description). The model considers all crucial macroscopic (i.e., sunlight absorption, emission, and nonradiative heat exchange) and microscopic processes (i.e., carrier generation, recombination and collection, as well as thermalization of hot generated carriers), in a broad wavelength range (e.g., 0.3–33 μm), i.e., considering both the solar wavelengths (0.3–4 μm), and the thermal wavelengths (≥ 4 μm), including the atmospheric transparency window (8–13 μm). To simulate the performance of our structures, we obtained the material parameters used for the active layer from Ref.^24^, of the electrodes from Refs.^16,25–29^ and of the other layers from Refs.^16,25,30^, see also Figs. S2 and S3 in the Supplementary Information.

**Figure 2:**
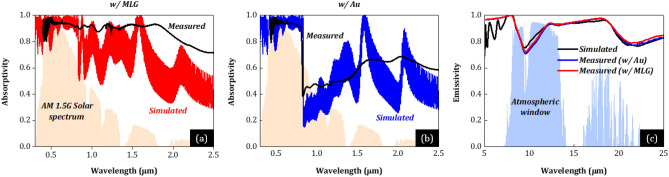
(**a, b**) Experimentally measured (black) and simulated (red and blue) solar absorptivity spectra of the two PSC samples w/MLG (red) and Au (blue), with the normalized AM1.5G solar spectrum plotted for reference (orange shaded area). Note that the PSC w/Au absorbs much less light of one-micron wavelength than the PSC w/MLG due to reflection from the device. (**c**) Simulated (black) and experimentally measured (red and blue) thermal emissivity spectra of the two PSC samples w/MLG (red) and Au (blue), with realistic atmosphere transmittance plotted for reference (blue shaded area).

We note that the absorption properties of PSCs in IR (~ *λ*_g_–4 μm) has not been examined in literature, despite seriously affecting *T*_c_ and PCE. To correctly account for the impact of filtering of IR light, we fabricated two PSC samples w/Au and MLG (see exact layer thicknesses for high PCE of fabricated PSCs in the Supplementary Information), characterized their solar absorption and thermal emission properties (see Figs. 2 and S1 in the Supplementary Information), and compared with our simulations (assuming same thicknesses with the experiments). Figures 2a,b show the measured (black) and simulated (red, blue) solar absorptivity spectra of the two PSC samples w/MLG and Au, respectively, with the normalized AM1.5G solar spectrum plotted for reference. Figure 2c shows the measured (red, blue) and simulated (black) thermal emissivity spectra of the two PSC samples, with realistic atmosphere transmittance plotted for reference. First at *λ* < *λ*_g_, the experimental (and simulated) absorptivity in both PSC cases is high and comparable (i.e., no matter Au or MLG), which is attributed to FAPbI_3_ of enhanced transport properties that allow for large perovskite layer thicknesses (< 800 nm—see Supplementary Information) and therefore efficient light absorption without lowering the EQE^18,19^. At *λ* > *λ*_g_, the experimental absorptivity (black) in both PSC cases validate the high NIR and SWIR absorption in state-of-the-art PSCs as predicted by the simulations (red/blue). Deviations in the absorptivity spectra, i.e., less pronounced absorptivity peaks (originating from interference in the thin film stack) and a systematic higher absorptivity of the characterized samples compared to simulations could also be attributed to the samples’ roughness, whereas in the simulations planar interfaces were assumed. For instance, the thicker MLG (~ 10 μm-thick compared to ~ 80 nm-thick Au) in general results to higher surface roughness (also one of the reasons for the lower *V*_OC_ output in PSCs w/MLG), which could be also the reason for the larger difference in absorptivity between simulation and experiment in case of “w/MLG” (Fig. 2a) compared to “w/Au” (Fig. 2b), especially in NIR and SWIR where perovskite does not absorb. Moreover, we note that a better agreement is expected if measured material properties (permittivity or refractive index of each material—see also Supplementary Information) are utilized^31^. According to simulation and experimental data, MLG strongly absorbs in NIR compared to Au, which acts as a reflector (see also Supplementary Information for more details), expected to lead to higher device heat load and therefore *T*_c_.

### Optical-electrical-thermal performance of MTE electrode.

**Figure 3:**
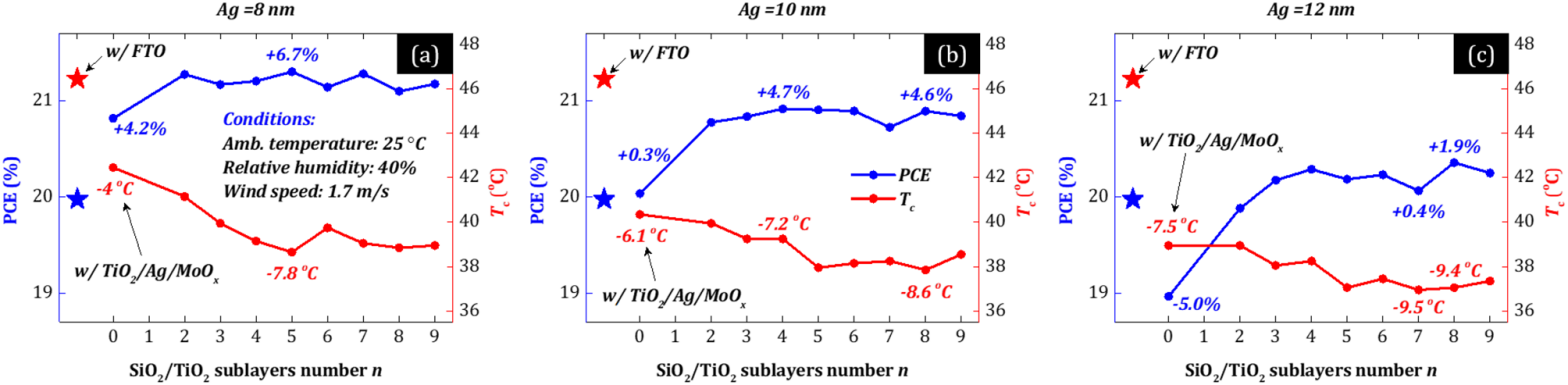
(**a–c**) Optimized PSCs’ operating temperature (*T*_c_—red) and power conversion efficiency (PCE—blue) as a function of multilayer electrodes’ SiO_2_/TiO_2_ sublayers number (*n*), for three Ag thickness of 8, 10, and 12 nm. Note that in all cases (*n* = 2–9), the first sublayers correspond to Al_2_O_3_/HfO_2_ for antireflection purposes (see Fig. 1a), while the case of *n* = 0 corresponds to a simple trilayer electrode w/TiO_2_/Ag/MoO_x_. *T*_c_ and PCE of the conventional PSC w/FTO are also plotted for reference (stars). The optimization of the PSCs was performed assuming typical weather conditions, i.e., 25 °C ambient temperature, 1.7 m/s wind speed, and 40% relative humidity.

Figures 3a–c show optimized PSCs’ *T*_c_ (red) and PCE (blue) as a function of multilayer electrodes’ SiO_2_/TiO_2_ sublayers number (*n*), for three Ag thickness of 8, 10, and 12 nm. PSCs’ optimization was performed assuming cells w/MLG. The comparable/high impact of MTE on cells w/Au is also shown in the next figures (see Figs. 6, 8) and compared to that w/MLG (see Figs. 5, 7). Note that in all cases (*n* = 2–9), the first sublayers correspond to Al_2_O_3_/HfO_2_ for antireflection purposes (see Fig. 1a), while the case of *n* = 0 corresponds to a simple trilayer electrode w/TiO_2_/Ag/MoO_x_. We also plot *T*_c_ and PCE of optimized PSC w/FTO (i.e., a conventional electrode) for reference (stars). The optimization of PSCs was performed assuming typical weather conditions, i.e., 1.7 m/s wind speed, 25 °C ambient temperature, and 40% relative humidity^32^. We also show later the impact of varying weather conditions. The red and blue inset numbers indicate the *T*_c_ decrease and relative PCE enhancement (%_rel._) compared to the conventional PSC w/FTO, corresponding to the TiO_2_/Ag/MoO_x_ trilayer electrode and the multilayer electrode with the highest PCE and the lowest *T*_c_.

First, the PCE of conventional PSCs w/MLG is ~ 20% and *T*_c_ ~ 46 °C, in agreement with experimental results^21,22,33^. Intuitively, integrating a composite electrode in a solar cell is thought to lead to low photocurrent and therefore low PCE because the compact metal film is reflective. Interestingly, the results in Fig. 3a indicate that the optimized PSCs w/MTE have high PCE up to ~ 21.3%, despite the compact 8 nm-thick Ag layer. This PCE is even higher (~ 6.7%_rel_) than that of conventional PSCs w/FTO (PCE ~ 20%). Even an MTE with a 12 nm-thick Ag can lead to higher PCE (~ 20.4%) than that of the conventional PSC case (see Fig. 3c). These results demonstrate that a composite electrode with compact metal film can result in high PCE, despite the general belief, through proper optimization of the total (electrode-cell) system.

An additional advantage of transparent composite structures with compact metal thin films is their ability to reflect the IR light substantially^6,8–11^, i.e., compared to other promising transparent thin-film electrodes such as metal nanowire networks (AgNWs)^34^ and carbon-based electrodes like carbon nanotubes (CNTs) and reduced graphene oxide (RGO)^35^. Therefore, they could simultaneously serve as compact IR filters to reduce the device heat load and *T*_c_ or for heat insulation in semi-transparent configurations. Interestingly, Fig. 3a–c shows that *T*_c_ (red) of the PSCs w/MTE is significantly lower than that of conventional PSCs w/FTO, up to ~ 9.5 °C depending on Ag thickness. Indicatively, a decrease in PSCs operating temperature by over ~ 9 degrees during outdoor operation (with common *T*_c_ > 50 °C^33^) may significantly enhance their operational stability^36^, leading to increased reliability/stability and higher system power output densities in the long term. These results demonstrate that MTE structures can substantially decrease a device heat load or used for efficient heat insulation/filtering for a variety of applications such as terrestrial solar cells or solar cells in space applications, semi-transparent and concentrated systems, and other electro-optical components including smart windows, light-emitting diodes, and displays.

Figure 3 also shows that the impact of adding more encapsulation layers on PSCs’ *T*_c_ and PCE improvement increases with Ag thickness. Specifically, PCE increases by adding more layers compared to the trilayer electrode (TiO_2_/Ag/MoO_x_) by 2.5, 4.4, and 6.9%_rel._ for an Ag thickness of 8, 10, and 12 nm, respectively. Interestingly though, even a MTE consisting of only three layers (TiO_2_/Ag/MoO_x_) can lead to high PCE ~ 20.8% (see Fig. 3a). We note that such performance (PCE > 20%) is the highest reported so far in solar cells with ultrathin-metal-film-based front contacts^2–4,11,13^.

**Figure 4:**
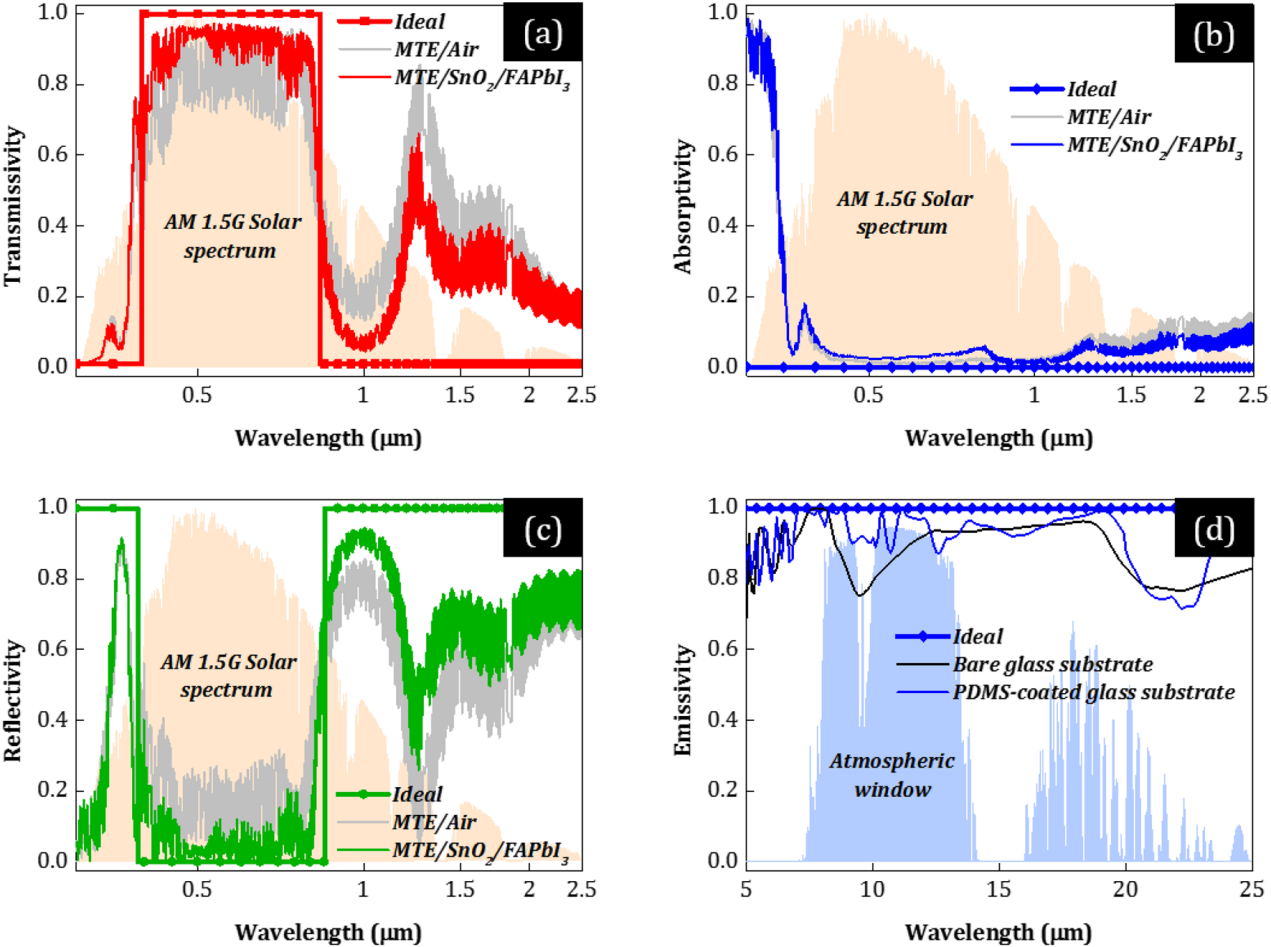
(**a–d**) Ideal (lines) solar reflectivity, transmissivity, absorptivity (0.3–2.5 μm), and thermal emissivity (> 4 μm) spectra for the FAPbI_3_-based PSC (i.e., with *λ*_g_ ~ 0.832 μm), compared to the ones calculated (curves) for the optimized multilayer electrode (MTE)-covered glass substrate (see Fig. 1a) that results to the highest PCE when integrated into the PSC (see *n* = 5 in Fig. 3a), together with the AM1.5G solar irradiance spectra and the infrared transmission of the atmosphere. To effectively calculate the transmitted light into the perovskite layer, we assume a SnO_2_ thin film and a semi-infinite FAPbI_3_ layer below MTE (i.e., PDMS/Glass/MTE/SnO_2_/FAPbI_3_). We also plot the case of air below MTE (i.e., PDMS/Glass/MTE/Air—gray) for comparison. The black curve in (**d**) corresponds to the thermal emissivity of the bare glass substrate (i.e., without PDMS) plotted for reference.

In Fig. 4, we show the physical origin of the high PCE (> 20%) and low *T*_c_ (< 40 °C) of PSCs w/MTE. Specifically, in Fig. 4a–d, we plot the solar reflectivity, transmissivity, and absorptivity/emissivity of the optimized MTE-covered glass substrate (Fig. 1a) resulting to the highest PCE when integrated into the PSC (see *n* = 5 in Fig. 3a). To effectively calculate the transmitted light into the perovskite layer, we assume a SnO_2_ thin film and a semi-infinite FAPbI_3_ layer below MTE (i.e., PDMS/Glass/MTE/SnO_2_/FAPbI_3_). For comparison, we also plot the case of air below MTE (i.e., PDMS/Glass/MTE/Air—gray). Lines correspond to the ideal reflectivity, transmissivity, and absorptivity/emissivity spectra for optimum (i) absorption in FAPbI_3_, (ii) filtering of UV and IR light, and (iii) radiative cooling, plotted for reference, and the curves to the spectral response of the optimized MTE-covered glass substrate.

First, Fig. 4a shows the origin of high PCE (> 20%). MTE exhibits high transparency (close to the ideal) in the beneficial wavelength spectrum where photons convert to photocurrent (~ 0.385–*λ*_g_ μm, *λ*_g_ ~ 0.832 μm for FAPbI_3_). From our calculations, the average optical transmittance (in ~ 0.385–*λ*_g_ μm) of the MTE-covered glass substrate is as high as ~ 91.4% despite the compact 8 nm-thick Ag layer, ~ 3.4% larger than that of the FTO-covered glass substrate (~ 88.0%) and very close (only ~ 2.9% lower) to that of the bare soda-lime glass substrate (94.3%). (In all cases, we assume PDMS and an optimized SnO_2_ thin film and semi-infinite FAPbI_3_ layer on the glass front and rear side, respectively, for a fair comparison.) This enhanced transmittance in the visible spectrum despite the reflective Ag layer is associated with reduced reflectance due to destructive interference of the reflected waves, where the reflectivity dip or transmission peak is controlled by the overcoat and undercoat film thicknesses. In our study, the addition of a higher number of optimized encapsulation layers leads to broader transmission band width along the visible spectrum and therefore improved transparency.

Figures 4b–d show the origin of low *T*_c_ (< 40 °C). First, the MTE with compact Ag exhibits broadband reflection of the IR radiation, further optimized in UV and NIR by the multilayer (Fig. 4c). Specifically, due to the integrated multilayer electrode with Ag, essentially a 1D PC, the MTE-covered glass substrate reflects 70% of the IR light, calculated from the simulated reflectivity in *λ*_g_–4 μm and the AM1.5G sun spectrum. In comparison, the FTO-covered glass substrate with conventional FTO reflects 14.9% (i.e., ~ 370% improvement). Additionally, the MTE-covered glass substrate parasitically absorbs 43.5 W/m^2^ of solar power (calculated from the simulated absorptivity in 0.3–4 μm and the AM1.5G sun spectrum) compared to 134.0 W/m^2^ of the conventional substrate due to the *R*_s_–*T* trade-off mitigation (see Fig. 4b), a significant improvement of ~ 68%. In MIR (> 4 μm), the thermal emissivity is almost maximum, especially within the atmospheric window (~ 8–13 μm), due to the addition of the PDMS film placed on top of the glass front side (blue curve), leading to black body-like radiative cooling (Fig. 4d). Specifically, the cooling power increases by 10 W/m^2^ [from 100.0 to 110.0 W/m^2^—by solving Eqs. (2) and (3)], an improvement of 10% compared to the bare glass substrate (black curve). We note that the photonic patterning of PDMS or glass may result in even higher cooling power, further decreasing solar cells’ *T*_c_^37,38^.

The results in Fig. 4 demonstrate that a composite electrode with compact metal film can result in high optical performance through proper optimization of the total (electrode-cell) system (see Fig. 3). Indicatively, assuming air below the MTE results to suboptimal light interference (gray curves in Fig. 4). Suboptimal light interference naturally leads to low photocurrent in solar cells. This effect is expected to be more intense in cases where the absorption or the thickness of the photoactive layer decreases, due to the more coherent light.

**Figure 5:**
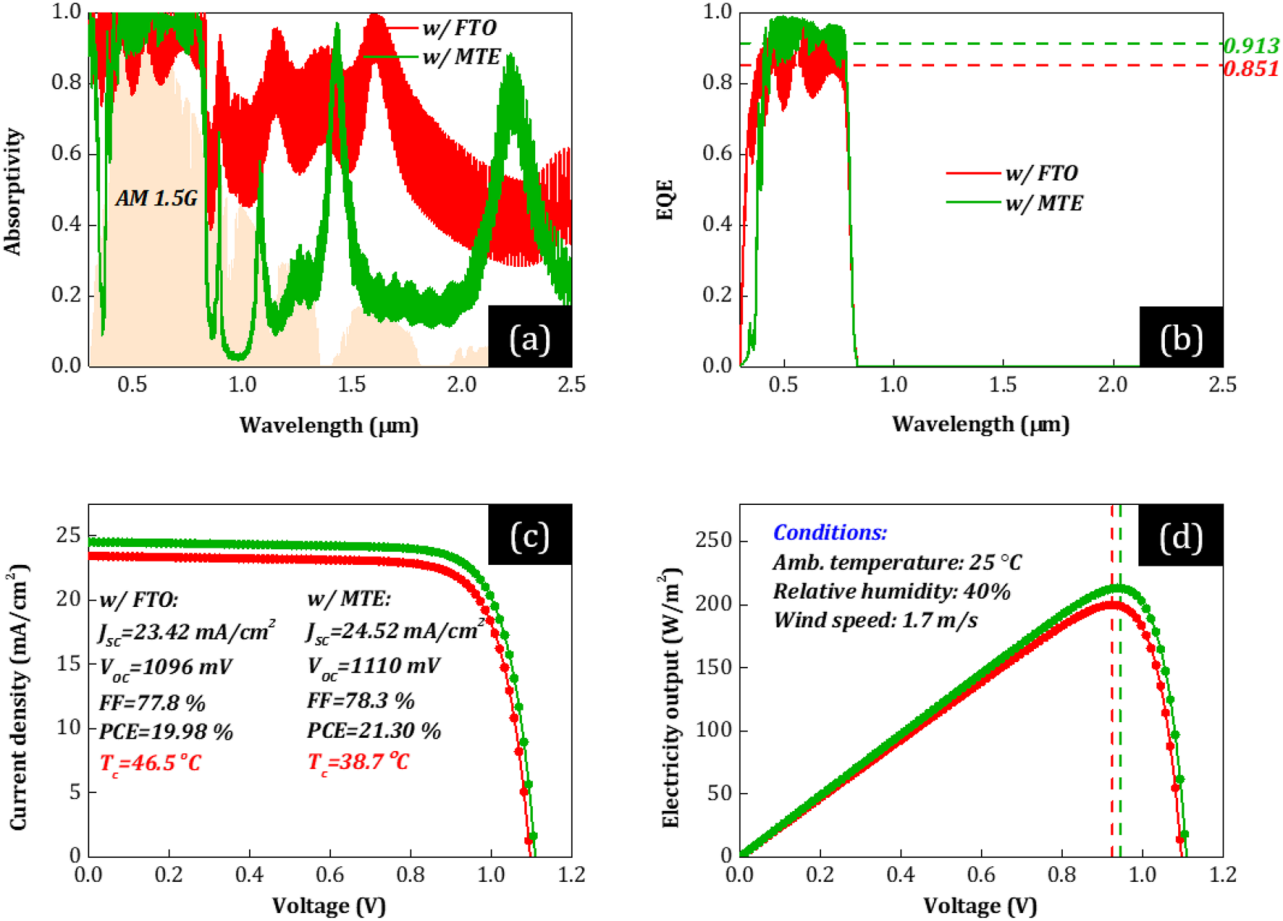
(**a**) Solar absorptivity, (**b**) external quantum efficiency (EQE), (**c**) *J*–*V* characteristics, and (**d**) power output of the optimized PSCs w/MLG and their corresponding PV characteristics (*J*_SC_, *V*_OC_, FF, and PCE) and cell operating temperature (*T*_c_), for common environmental conditions, i.e., 25 °C ambient temperature, 1.7 m/s wind speed, and relative humidity RH = 40%. The green curves correspond to the PSC w/MTE (*n* = 5 and Ag = 8 nm) and the red w/FTO. The horizontal dashed lines in (**b**) correspond to the average EQE (at 0.4–0.8 μm) of each PSC. The vertical dashed lines in (**d**) correspond to the output voltage at the maximum power point (mpp) of each PSC. Both PSCs correspond to the optimum devices optimized according to the optimization procedure discussed in the main text.

**Figure 6:**
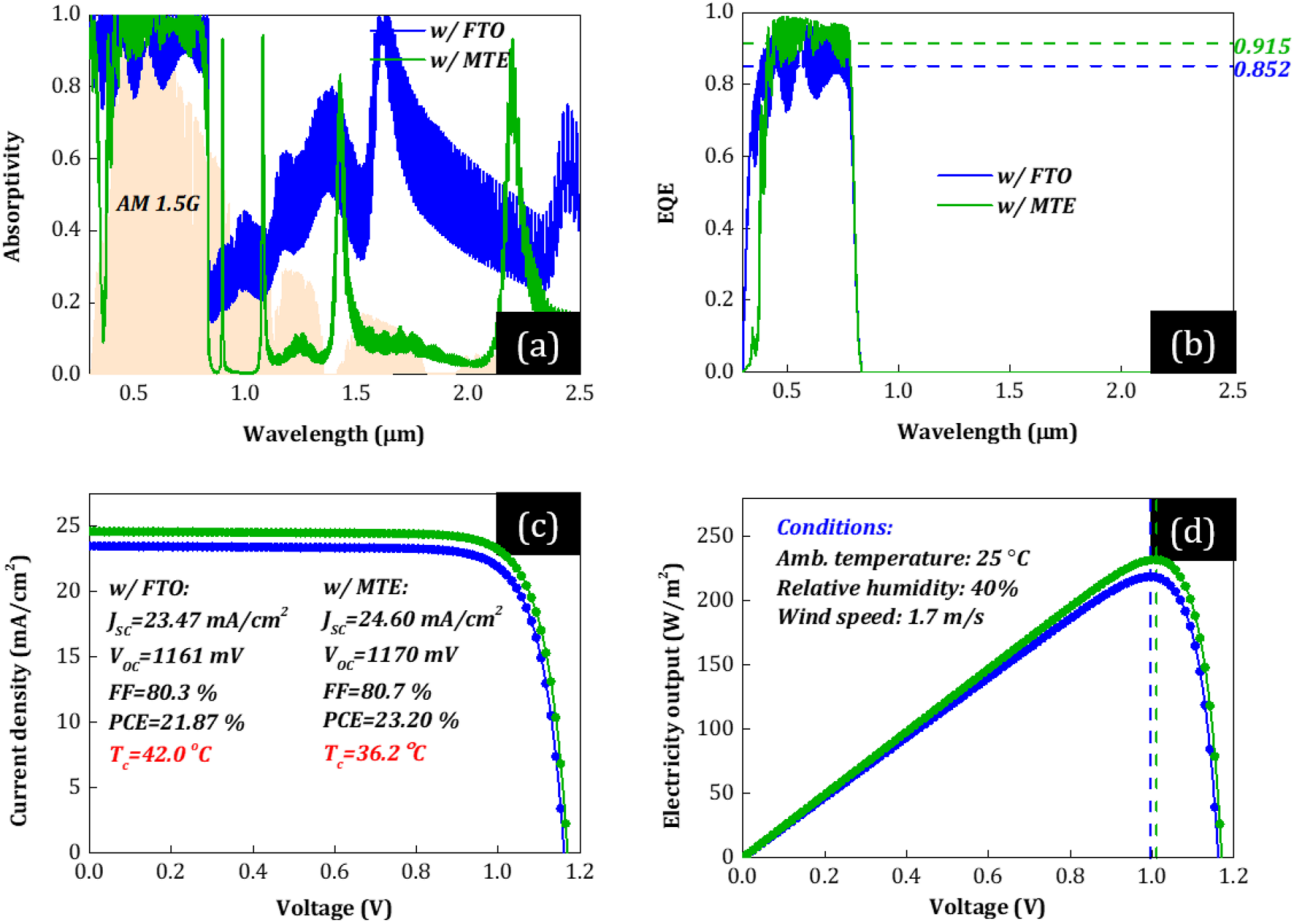
(**a**) Solar absorptivity, (**b**) external quantum efficiency (EQE), (**c**) *J*–*V* characteristics, and (**d**) power output of the optimized PSCs w/Au and their corresponding PV characteristics (*J*_SC_, *V*_OC_, FF, and PCE) and cell operating temperature (*T*_c_), for common environmental conditions, i.e., 25 °C ambient temperature, 1.7 m/s wind speed, and relative humidity RH = 40%. The green curves correspond to the PSC w/MTE (*n* = 5 and Ag = 8nm) and the blue w/FTO. The horizontal dashed lines in (**b**) correspond to the average EQE (at 0.4–0.8 μm) of each PSC. The vertical dashed lines in (**d**) correspond to the output voltage at the maximum power point (mpp) of each PSC. Both PSCs correspond to the optimum devices optimized according to the optimization procedure discussed in the main text.

With 91.4% visible light transmitted, providing ~ 3.9% improvement, 70% infrared solar radiation reflected, providing ~ 370% improvement, and 4.4% solar radiation absorbed, providing ~ 68% improvement, integrating the MTE-covered glass substrate in solar cells can significantly affect their spectral properties and therefore their electrical and thermal response. In Figs. 5a–b and 6a–b, we show the absorptivity and external quantum efficiency (EQE) of the optimized PSCs w/MTE (green) corresponding to the highest PCE (see *n* = 5 in Fig. 3a), assuming cells w/MLG and Au, respectively. Note that the thicknesses of each layer of the MTE-cell are optimized for each different cell case (i.e., w/MLG and w/Au), while for both cases *n* = 5 and Ag = 8 nm. We also plot the case of the optimized PSCs with conventional FTO for comparison, assuming cells w/MLG (red) and Au (blue).

In IR (~ *λ*_g_–4 μm), both conventional PSCs (w/FTO) shows strong light absorption (red and blue in Fig. 5a and 6a, respectively) despite IR photons’ lower energy than photoactive material’s band gap energy. The reason is that FTO and MLG strongly absorb in NIR, in agreement with the fabricated PSCs (see Fig. 2a,b). Moreover, conventional PSC w/MLG (red) shows an even stronger absorption in IR than w/Au (blue). The reason is that MLG strongly absorbs in NIR compared to Au, which acts as a reflector. This absorption does not contribute to photocurrent and therefore is expected to lead to excess heat generation and high *T*_c_ in realistic outdoor conditions. Specifically, the device thermal load equals to 611.7 (w/MLG) and 501.1 W/m^2^ (w/Au) calculated by the total sun power (AM1.5G) minus the reflected and electrical power output of the PSCs at the steady state [by solving Eq. (1)]. In contrast, PSCs w/MTE show very low absorption of the IR light (green in Figs. 5a and 6a), due to strong reflection from the MTE (see Fig. 4c) and its low thickness that result to low parasitic absorption in the device (see Fig. 4b). The device thermal load equals to 434.6 (w/MLG) and 372.2 W/m^2^ (w/Au), i.e., significantly lower (by 177.1 and 128.9 W/m^2^, respectively) than that of the conventional PSCs w/FTO (611.7 and 501.1 W/m^2^, respectively). In the beneficial wavelength range (0.385–*λ*_g_ μm), all PSCs show strong light absorption, as expected. The PSCs w/MTE (green) show even higher solar absorption. As shown in Figs. 5b and 6b, where we plot PSCs’ EQE, this higher solar absorption results from improved absorption in perovskite, which is attributed to improved impedance matching and antireflection induced by the PDMS film and the multilayer electrode, expected to lead to higher photocurrent.

The improved optical response of the PSCs w/MTE (green) compared to the conventional PSCs w/FTO (red, blue) (see Figs. 5a–b and 6a–b) is expected to lead to improved electrical and thermal response. In Figs. 5c–d and 6c–d, we plot PSCs’ *J*-*V* and power output for an operating or cell temperature equal to the steady-state temperature calculated by solving Eq. (1). First, the output current density increases mainly due to the improved short-circuit current density (*J*_SC_) (see green versus red and blue curves in Figs. 5c and 6c, respectively) obtained by integrating EQE (Figs. 5b and 6b) over the AM1.5G spectrum [see Eq. (8)].* J*_SC_ equals to ~ 23.4 and ~ 24.5 mA/cm^2^ for the optimized PSCs w/FTO and w/MTE, respectively, an increase of ~ 4.7% in relative terms. We note that the comparable *J*_SC_ in cells w/MLG and Au is attributed to the enhanced transport properties of FAPbI_3_ that allow perovskite layer thicknesses as large as 800 nm for efficient light absorption without lowering the EQE^18,19,39^. Specifically, the optimized PSC cases w/FTO and MTE correspond to ~ 690- and ~ 650 nm-thick FAPbI_3_, respectively, leading to efficient light absorption at *λ* < *λ*_g_ (see Figs. 5a–b and 6a–b). In contrast, the open-circuit voltage, *V*_OC_, [i.e., solving Eq. (7) for *J* = 0] of PSCs w/MLG is lower than that of PSCs w/Au (see red curve in Fig. 5c vs. blue curve in Fig. 6c for *J* = 0), leading to lower power output (see Figs. 5d vs. 6d), in agreement with literature^21,22^. Moreover, despite the increased solar absorption in 0.385–*λ*_g_ μm in PSCs w/MTE than the conventional PSCs w/FTO (and hence the associated increased parasitic and thermalization losses that increase heat dissipation in the structure), at the steady state [by solving Eq. (1)], the enhanced reflection of IR light and radiative cooling result in lower *T*_c_ (see inset captions in Figs. 5c and 6c), which further affect the *J*–*V* characteristics. Specifically, with a decrease in *T*_c_, the carrier concentration decreases exponentially [see Eq. (11)], leading to a lower dark current density [see Eq. (10)]. *V*_OC_ increases exponentially with a decrease in the dark current at lower *T*_c_, leading to a temperature impact on PSCs’ energy yield and a negative PSC power-temperature coefficient (*β*—see Table 1 in the Methods Section). Therefore, the output voltage also increases due to the *T*_c_ reduction and *V*_OC_ rise, which is more evident in the case of the PSCs w/MLG due to the higher decrease of *T*_c_ [see higher output voltage values of PSCs w/MTE (green) than those of conventional PSCs w/FTO (red/blue) in Figs. 5d and 6d at the maximum power point (mpp—dashed lines)]. We note that besides *T*_c_ reduction, an increase in *J*_SC_ is also expected to impact *V*_OC_ by means of shifting the *J*–*V* curve upwards according to the superposition principle. This impact is expected to be lower in the case of already optically optimized solar cells^40^. In our case, the relative enhancement of *J*_SC_ by ~ 4.7% (due to improved antireflection) was calculated to constitute ~ 15% of the overall *V*_OC_ improvement. Eventually, due to the *J*_SC_, *V*_OC_, and FF increase, the power conversion efficiency [PCE(*V*_mp_,*T*_c_) = *J*_SC_*V*_OC_(*T*_c_)*FF*(*T*_c_)/∫*I*(*λ*)d*λ* = *J*(*V*,*T*_c_)*V*(*T*_c_)|_mp_/∫*I*(*λ*)d*λ*] increases by 6.6 (w/MLG) and 6.1% (w/Au), in relative terms, assuming typical weather conditions, i.e., 1.7 m/s wind speed, 25 °C ambient temperature, and 40% humidity.

**Figure 7:**
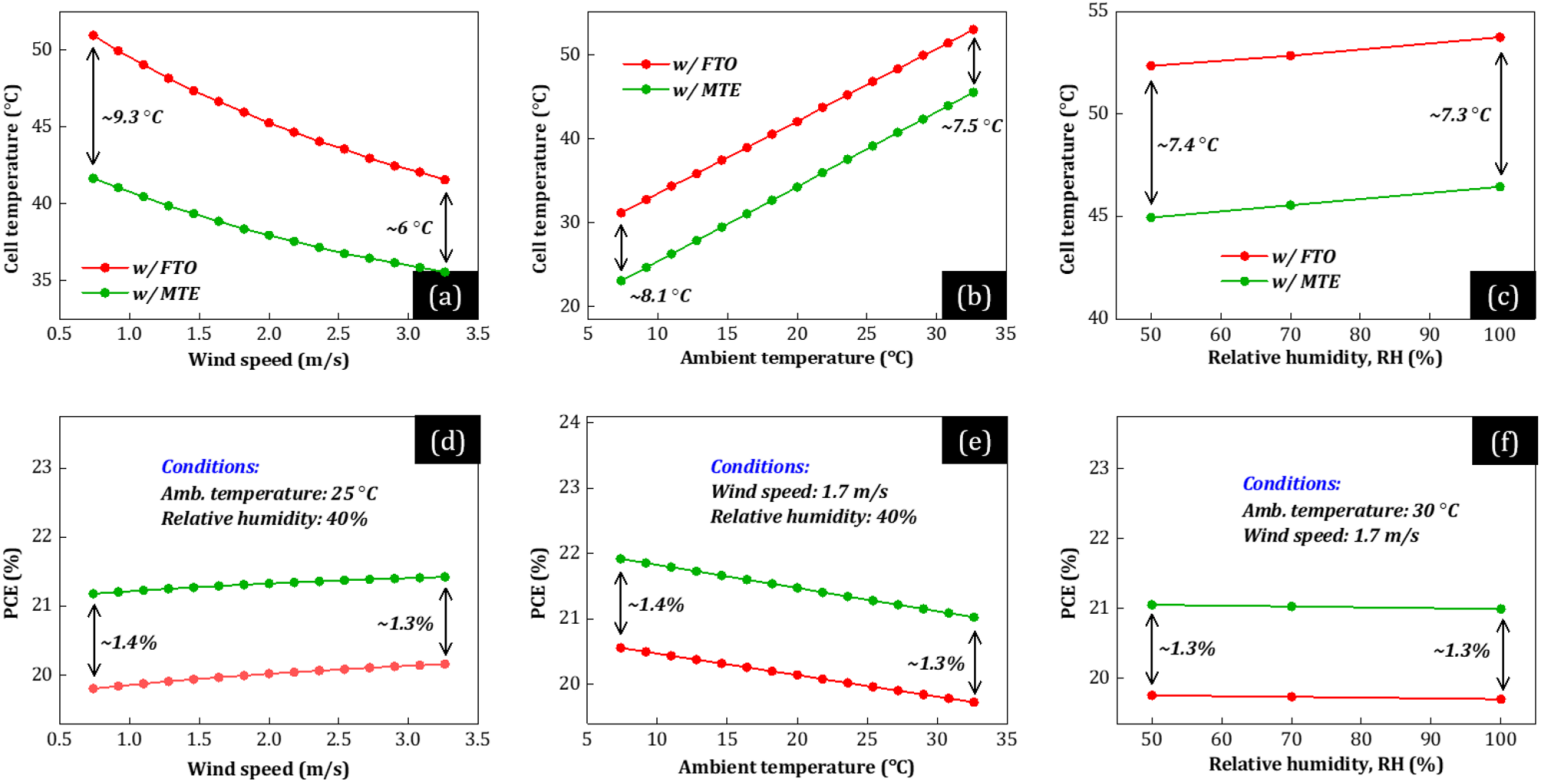
(**a–c**) Cell operating temperature (*T*_c_) and (**c–d**) power conversion efficiency (PCE) of the optimized PSCs w/MLG as a function of (**a, d**) wind speed, (**b, e**) ambient temperature, and (**c, f**) relative humidity. The green curves correspond to the PSC w/MTE (*n* = 5 and Ag = 8 nm) and the red w/FTO.

**Figure 8:**
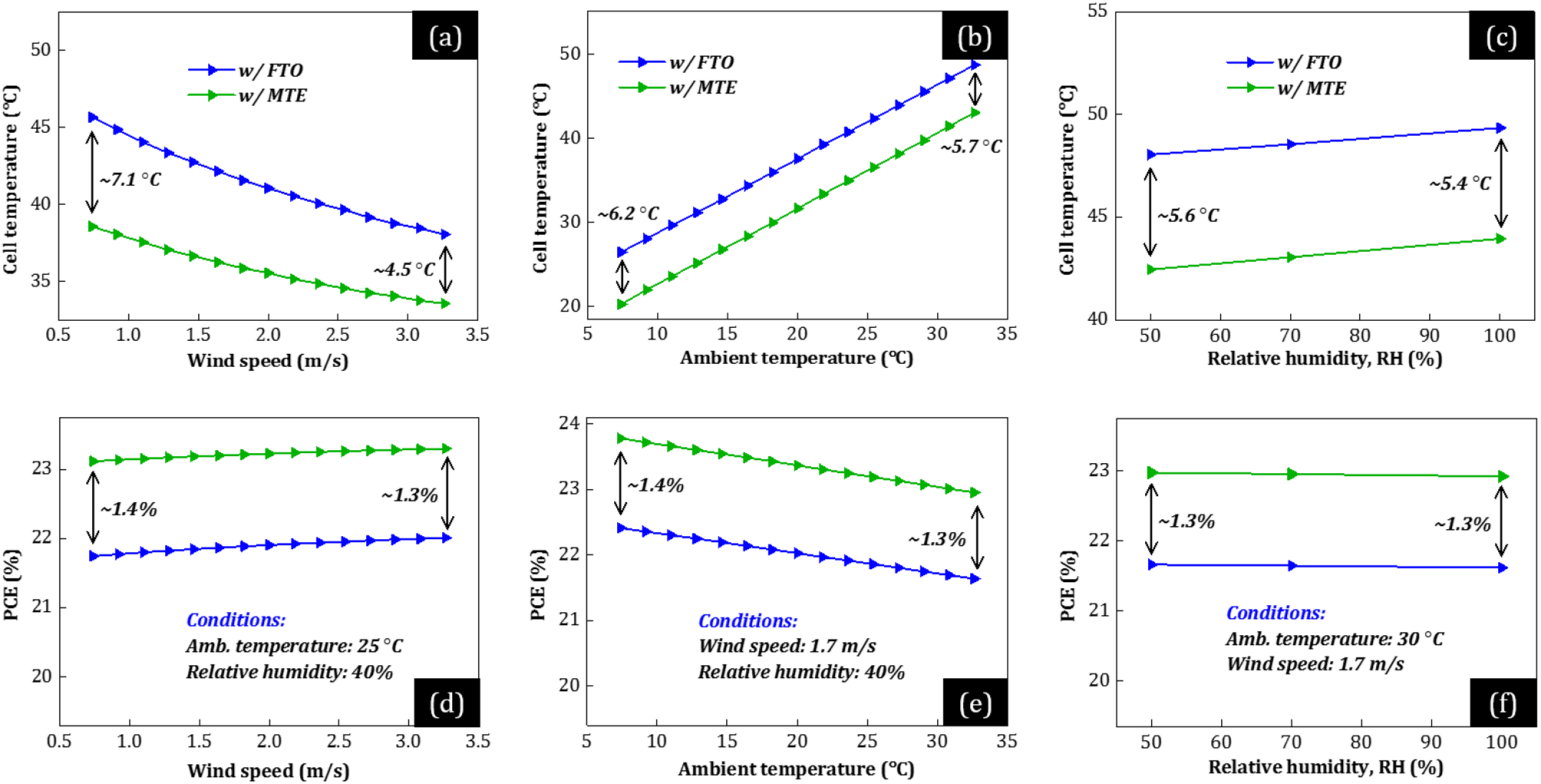
(**a–c**) Cell operating temperature (*T*_c_) and (**c–d**) power conversion efficiency (PCE) of the optimized PSCs w/Au as a function of (**a, d**) wind speed, (**b, e**) ambient temperature, and (**c, f**) relative humidity. The green curves correspond to the PSC w/MTE (*n* = 5 and Ag = 8 nm) and the blue w/FTO.

Figures 7 and 8 show the practical benefit of composite electrodes in realistic conditions. Specifically, we plot *T*_c_ (a–c) and PCE (d–f) of optimized PSCs w/MLG (Fig. 7) and w/Au (Fig. 8) as a function of varying environmental conditions, i.e., wind speed (a, d), ambient temperature (b, e), and relative humidity (c, f). The green curves correspond to the optimized PSCs w/MTE and the red, blue curves to the optimized PSCs w/FTO.

First, our theoretically calculated PCE (d–f) and *T*_c_ (a–c) of conventional PSCs w/FTO (red, blue) may reach values of ~  > 20% and up to ~ 50 °C, respectively, in agreement with experimental studies^21,22,33^, comparable to bulkier silicon-based counterparts (~ 250 μm thickness)^32^. Moreover, the conventional PSC w/MLG (red) operates at 3.5 to 5.3 °C higher *T*_c_ than w/Au (blue). The reason is the increased solar heating power, originating from the increased parasitic absorption in MLG compared to Au, mainly in NIR (see Figs. 5a and 6a). Additionally, the conventional PSC w/MLG (red) exhibits lower absolute PCE by up to 3% than w/Au (blue), mainly due to *V*_OC_-loss due to the HTL-MLG interface (see Fig. 1b). Notably, PSCs w/MTE (green) operate at much lower *T*_c_ [up to 9.3 (w/MLG) and 7.1 °C (w/Au)] than conventional PSCs w/FTO (red, blue) and provide higher PCE (+ 1.3%). This difference in *T*_c_ and PCE is mainly not affected by the environmental conditions. Specifically, *T*_c_ of PSCs w/MTE is 6.0 to 9.3 °C (w/MLG) and 4.5 to 7.1 °C (w/Au) lower than that of conventional PSCs w/FTO, no matter wind speed, ambient temperature, and humidity (a–c), whereas their PCE is 1.3 to 1.4% higher (d–f). Additionally, in extreme environmental conditions, i.e., low wind speed (see Figs. 7a and 8a), where *T*_c_ should be as low as possible to avoid permanent damage, the cell temperature difference is the highest and over 9 (w/MLG) and 7 °C (w/Au). Indicatively, such a temperature decrease (with common *T*_c_ > 50°C^33^) may significantly enhance the operational stability of PSCs^36^, revealing the high potential of composite electrodes with metal thin films in high-efficiency optoelectronic devices such as solar cells, semi-transparent, and concentrated systems.

## Conclusions

We have demonstrated a comprehensive EM analysis and guidelines for enhancing the optical efficiency of ultrathin-metal-based-film transparent conductive electrodes such as TCO–metal–TCO, dielectric-metal-dielectric (DMD) or insulator–metal–insulator (IMI) composite structures. A detailed numerical analysis on their antireflection properties as a function of materials and layers number when integrated in complex-multilayer configurations (as those of solar cells) showed the origin and potential of enhanced transparency and filtering. The thorough investigation demonstrated the importance of metal encapsulation layers’ number and material for optimal light interference and broadband response. To assess the application potentials, *J*_ph_ > 25 mA/cm^2^, PCE > 20%, and vastly reduced device heat load by 177.1 W/m^2^ was achieved when integrated in state-of-the-art perovskite solar cells, revealing the high potential of composite electrodes with metal thin films in high-efficiency optoelectronic devices such as solar cells, semi-transparent, and concentrated systems.

## Methods

### Perovskite solar cells fabrication

Fluorine-doped tin oxide (FTO) glass substrates (TCO glass, TEC8) were etched using Zn powder and diluted hydrochloric acid (HCl), cleaned by ultrasonication in Hellmanex (2%, deionized water), deionized water, acetone, and ethanol. After drying the substrates with a nitrogen gun, they were UV-O_3_ treated for 15 min. Afterwards, an approximately 20 nm thick blocking layer (TiO_2_) was deposited on the FTO by spray pyrolysis at 450 °C using a commercial titanium diisopropoxide bis(acetylacetonate) solution (75% in 2-propanol, Sigma-Aldrich) diluted in anhydrous ethanol (1:9 volume ratio) as a precursor and oxygen as a carrier gas. A mesoporous TiO_2_ layer was deposited by spin-coating a diluted paste (Dyesol 30NRD) in ethanol (1:6 weight ratio) at 4000 rpm for 15 s and sintering at 450 °C for 30 min in a dry-air atmosphere. The perovskite films were deposited from the precursor solution, which was prepared in an argon atmosphere by dissolving FAI, MABr, PbI_2_ and PbBr_2_ in anhydrous dimethylformamide/dimethyl sulfoxide (4:1 volume ratio) to achieve the desired compositions (FAPbI_3_)_0.98_(MAPbBr_3_)_0.02_ using a 3% PbI_2_ excess and 44 mg of MACl. The perovskite precursor was deposited in a dry-air atmosphere on FTO/c-TiO_2_/m-TiO_2_ substrate, using a single-step deposition method (6000 rpm for 50 s). To control the film crystallization, 10 s before the end of the spin-coating program, the perovskite precursor was quenched with chlorobenzene as the antisolvent. To form and crystallize the perovskite, the spin-coated perovskite precursors were annealed at 150 °C for 30 min inside a dry-air atmosphere. Subsequently, the perovskite films were then passivated by spin-coating (6000 rpm for 50 s) a 3 mg mL^−1^ dispersion of octylamonium iodide (OAI) in isopropanol. The HTM (Spiro-OMeTAD doped with bis(trifluoromethylsulfonyl)imide lithium salt (17.8 μL of a solution of 520 mg of LiTFSI in 1 mL of acetonitrile) and 28.8 μL of 4-*tert*-butylpyridine)) was deposited by spin-coating at 4000 rpm for 30 s. Finally, an approximately 80 nm gold (Au) layer or 10 μm multi-layer Graphene layer (homogenized Timrex KS25 powders), were deposited by thermal evaporation and doctor-blade coating, respectively.

### Perovskite solar cells characterization

Fourier-transform infrared spectroscopy (FT-IR) measurements were carried out under vacuum, with a Bruker Vertex 70v FT-IR vacuum spectrometer (Bruker Optik GmbH, Rosenheim, Germany); The transmission of the samples was evaluated using a PIKE universal sample holder (PIKE Technologies, Inc.—Madison, USA), while reflection was measured using a Bruker Optics A513 reflection accessory (Bruker Optik GmbH, Rosenheim, Germany), at an angle of incidence of 7 degrees. To cover a spectral range of 0.45–25 μm, two different sets of optics were used: (a) for 0.45–1.25 μm, a Quartz beamsplitter and a room temperature Silicon diode detector, while (b) for 1.3–25 μm), a broad band KBr beamsplitter and a room temperature broad band triglycine sulfate (DTGS) detector were used. In any case, interferograms were collected at 4 cm^-1^ resolution (8 scans), apodized with a Blackman-Harris function, and Fourier transformed with two levels of zero filling to yield spectra encoded at 2 cm^−1^ intervals. Before scanning the samples, an empty holder and an aluminum mirror (> 90% average reflectivity) background measurement was recorded in vacuum for transmission and reflection measurements, respectively, and each sample spectrum was obtained by automatic subtraction of it.

### Opto-electro-thermal calculation of perovskite solar cells

We perform a combined thermal-optical-electrical analysis to calculate PCE as a function of *T*_c_. First, we calculate the absorbed solar power in the encapsulated PSCs based on the simulated PSCs absorptivity, by employing the transfer matrix method, and use it as the heat input in the electro-thermal simulation. We then set up a coupled electro-thermal simulator solving the steady-state energy balance for solar cells, with which we simulate the cell operating temperature (*T*_c_) and the power conversion efficiency (PCE), assuming varying ambient temperature, humidity, and wind speed to mimic typical outdoor conditions^16,41^:

1 $$P_{{\text{r}}} \left( {T_{{\text{c}}} } \right) + P_{{\text{c}}} \left( {T_{{\text{c}}} ,T_{{\text{a}}} } \right) + P_{{\text{g}}} \left( {T_{{\text{c}}} ,T_{{\text{a}}} } \right) = P_{{\text{h}}} \left( {V_{{{\text{mp}}}} ,T_{{\text{c}}} } \right) + P_{{\text{a}}} \left( {T_{{\text{a}}} } \right),$$

In Eq. (1), *P*_h_(*T*_c_) is the heat flux from solar radiation and *P*_a_(*T*_a_) is the radiative heat flux from the atmosphere, absorbed by the device at ambient temperature, *T*_a_. *P*_r_(*T*_c_) is the total heat flux radiated by the solar cell at *T*_c_, *P*_c_(*T*_c_, *T*_a_) accounts for the outgoing nonradiative heat transfer, and *P*_g_(*T*_c_, *T*_a_) is the radiative heat flux by the rear surface of the solar cell. These power terms are given by^15,16^

2 $$P_{{\text{r}}} \left( {T_{{\text{c}}} } \right) = \int_{0}^{\infty } {\int_{0}^{2\pi } {\int_{0}^{\pi /2} {I_{{{\text{BB}}}} \left( {\lambda ,T_{{\text{c}}} } \right)\varepsilon \left( {\lambda ,\theta ,\varphi } \right){\text{cos}}\theta \,{\text{sin}}\theta \,{\text{d}}\theta \,{\text{d}}\varphi \,{\text{d}}\lambda } } } ,$$

3 $$P_{{\text{a}}} \left( {T_{{\text{a}}} } \right) = \int_{0}^{\infty } {\int_{0}^{2\pi } {\int_{0}^{\pi /2} {I_{{{\text{BB}}}} \left( {\lambda ,T_{{\text{a}}} } \right)\varepsilon \left( {\lambda ,\theta ,\varphi } \right)\varepsilon_{{\text{a}}} \left( {\lambda ,\theta } \right){\text{cos}}\theta \,{\text{sin}}\theta \,{\text{d}}\theta \,{\text{d}}\varphi \,{\text{d}}\lambda } } }$$

4 $$P_{{\text{g}}} = \sigma \varepsilon_{{\text{r}}} A\left( {T_{{\text{c}}}^{4} - T_{{\text{a}}}^{4} } \right),$$

5 $$P_{{\text{c}}} \left( {T_{{\text{c}}} ,T_{{\text{a}}} } \right) = h_{{\text{c}}} \left( {T_{{\text{c}}} - T_{{\text{a}}} } \right),$$

6 $$P_{{\text{h}}} \left( {V_{{{\text{mp}}}} ,T_{{\text{c}}} } \right) = \int_{0}^{\infty } {I\left( \lambda \right)\varepsilon \left( \lambda \right)d\lambda - PCE\left( {V_{{{\text{mp}}}} ,T_{{\text{c}}} } \right)\int_{0}^{\infty } {I\left( \lambda \right){\text{d}}\lambda } ,} { }$$

where *λ* is the free-space wavelength, *σ* is the Stefan-Boltzmann constant, *A* ~ 1 is the view factor, *I*_BB_(*λ*, *T*_c_) is the spectral intensity of a blackbody at temperature *T*_c_ given by Planck’s law, *I*(*λ*) is the solar illumination represented by the measured sun’s radiation, the AM1.5G spectrum, and *h*_c,top_ and *h*_c,bottom_ are the wind-speed-dependent nonradiative heat transfer coefficients (higher *h*_c_ values correspond to stronger winds) at the top and rear surfaces of the solar cell, respectively. For *h*_c,top_ and *h*_c,bottom_, we use two relations, frequently used in previous studies for similarly encapsulated solar cell systems, expressed as *h*_c,top_ = 5.8 + 3.7*v*_w_ and *h*_c,bottom_ = 2.8 + 3.0*v*_w_, where *v*_w_ is the velocity of wind at the module surface (in m/s) given by the relationship suggested in the literature *v*_w_ = 0.68*v*_f_ − 0.5, where *v*_f_ is the wind speed measured by the closest weather station^32^. *ε*(*λ*, *θ*, *φ*) is the solar cell spectral directional emissivity (equal to absorptivity, according to Kirchhoff’s law), *ε*_a_(*λ*,*θ*) = 1 − *t*(*λ*)^1/cos*θ*^ is the angle-dependent emissivity of the atmosphere, with *t*(*λ*) the atmospheric transmittance in the zenith direction, and *ε*_r_ ~ 0.85 is the solar cell rear surface hemispherical emissivity^16^. Due to energy conservation, *P*_h_ equals the difference between absorbed solar energy flux and generated electrical power in the solar cell, where PCE(*V*_mp_,*T*_c_) = *J*(*V*,*T*_c_)*V*(*T*_c_)|_mp_/∫*I*(*λ*)d*λ* is the temperature-dependent cell’s solar-to-electrical power conversion efficiency (PCE) assuming that it operates at its maximum power point (mp)^16^, where *J* and *V* are the output current density and voltage, respectively. In Eq. (6), we assume that the structure is facing the sun at a fixed angle. Thus, the term *P*_h_ does not have an angular integral, and solar cell’s absorptivity/emissivity is represented by its value at normal incidence.

In the present study, we assume dominating recombination by the space charge region since most of the perovskite layer is depleted^42^. Assuming that Shockley–Read–Hall recombination is the dominant nonradiative recombination mechanism and in the presence of shunt resistance (accounting for manufacturing defects and impurities near the junction), we calculate the current–voltage characteristics by the following diode equation^15,16,43^:

7 $$J\left( {V,T_{{\text{c}}} } \right) = J_{{{\text{SC}}}} - J_{{{\text{r}},0}} \left( {T_{{\text{c}}} } \right)\left( {e^{{\left( {qV/k_{{\text{B}}} T_{{\text{c}}} } \right)}} - 1} \right) - J_{{{\text{nr}},0}} \left( {V,T_{{\text{c}}} } \right)\left( {e^{{\left( {qV/2k_{{\text{B}}} T_{{\text{c}}} } \right)}} - 1} \right) - \frac{V}{{R_{{{\text{sh}}}} }},$$

where *q* is the elementary charge of an electron, *k*_B_ is Boltzmann’s constant, and *R*_sh_ is the solar cell shunt resistance. The term

8 $$J_{{{\text{SC}}}} = q\int_{0}^{{\lambda_{{{\text{BG}}}} }} {I\left( \lambda \right)EQE\left( \lambda \right){\text{d}}\lambda } ,$$

is the current density flowing at short-circuit conditions under the sun illumination, where *EQE*(*λ*) is the external quantum efficiency of the solar cell. *EQE*(*λ*) is defined as the multiplication of the internal quantum efficiency [i.e., number of charge carriers collected versus the number of absorbed photons—*IQE*(*λ*)] and the absorption efficiency of the active layer, *ε*_al_(*λ*), i.e., *EQE*(*λ*) = *IQE*(*λ*)*ε*_al_(*λ*). *IQE*(*λ*) is extracted from Ref.^44^ and is close to unity, mainly due to the low thickness of the active layer. The second and the third terms correspond to the radiative and nonradiative recombination current densities, respectively, with the corresponding dark-saturation current densities *J*_r,0_ and *J*_nr,0_, given by Eqs. (9) and (10), and ideality factors of 1 and 2, respectively:

9 $$J_{{{\text{r}},0}} \left( {T_{{\text{c}}} } \right) = q\int_{0}^{{\lambda_{{{\text{BG}}}} }} {I_{{{\text{BB}}}} \left( \lambda \right)\varepsilon_{{{\text{al}}}} \left( \lambda \right){\text{d}}\lambda ,} { }$$

10 $$J_{{{\text{nr}},0}} \left( {V,T_{{\text{c}}} } \right) \propto \frac{{n_{{\text{i}}} \left( {T_{{\text{c}}} } \right)}}{\tau }\sqrt {\left( {V_{{{\text{bi}}}} \left( {T_{{\text{c}}} } \right) - V} \right)} ,{ }$$

where *τ* = 1 μs is the lifetime of electrons and holes (assuming equal lifetimes for electrons and holes) extracted from Ref.^45^, *V*_bi_(*T*_c_) is the temperature-dependent built-in bias, and *n*_i_(*T*_c_) is the temperature-dependent intrinsic charge carrier density. Since the built-in bias is typically slightly higher than the open-circuit voltage, we set it a bit higher than the open-circuit voltage of the Shockley-Queisser limit for perovskite material’s bandgap^43^. The density of states in the conduction, *N*_C_, and the valence band, *N*_V_, assuming *N*_C_ = *N*_V_, are extracted by DFT calculations^46^. We calculate then the temperature-dependent intrinsic charge carrier density by

11 $$n_{{\text{i}}} \left( {T_{{\text{c}}} } \right) = 2\left( {\frac{{2\pi mk_{{\text{B}}} T_{{\text{c}}} }}{{h^{2} }}} \right)^{3/2} e^{{ - E_{{\text{g}}} \left( {T_{{\text{c}}} } \right)/2k_{{\text{B}}} T_{{\text{c}}} }} ,$$

where *h* is Planck’s constant, *m* is the effective mass of the electrons and holes (assuming equal electrons’, holes’ effective mass), and *E*_g_(*T*_c_) is the temperature-dependent bandgap, which is assumed to increase by 0.35 meV per 1 K^47^. We fit our model to the current–voltage characteristics of the examined promising PSCs w/MLG and w/Au back contacts^19,22^. The PV characteristics at 25 °C and 1000 W/m^2^ of solar radiation, i.e., short-circuit current density, *J*_SC_, open-circuit voltage, *V*_OC_, (i.e., for *J* = 0), fill factor, *FF* = *J*(*V*)*V*|_mp_/*J*_SC_*V*_OC_, and output power-temperature coefficient (*β*) are summarized in Table 1.

**Table 1:** PV characteristics at 25 °C and 1000 W/m^2^ of solar radiation, i.e., short-circuit current density, *J*_SC_, open-circuit voltage, *V*_OC_, (i.e., for *J* = 0), fill factor, *FF* = *J*(*V*)*V*|_mp_/*J*_SC_*V*_OC_, PCE, and output power-temperature coefficient (*β*).

PSC	*V*_OC_ (V)	*J*_SC_ (mA/cm^2^)	*FF* (%)	PCE (%)	*β* (%/°C)
w/MLG	1.12	22.66	78.9	20.06	− 0.25
w/Au	1.18	25.50	81.2	24.53	− 0.25

To evaluate the validity of the modeled solar cell’s temperature dependence, we compare our calculated power-temperature coefficients [i.e., the slopes of the *PCE* (%)–*T*_c_ curves] with those in literature [the slopes of the *PCE* (%)–*T*_c_ curves are normalized at % compared to the solar cells operating at Standard Test Conditions (STC) (i.e., 1000 W/m^2^ of solar radiation, *T*_c_ = 298.15 K)]. The calculated power-temperature coefficients (*β*) of the next-generation perovskite-based solar cells are equal to − 0.25%/°C, in agreement to literature reports calculated from experimental data for solar cells’ typical operating temperatures range^33^.

### Supplementary Information

Supplementary Information.

## Data Availability

The datasets used and/or analyzed during the current study are available from the corresponding authors on reasonable request.
