# Supplementary material for: Infrared-reflective ultrathin-metal-film-based transparent electrode with ultralow optical loss for high efficiency in solar cells
Source: Sci Rep. 2024 Jan 4;14:548. doi: 10.1038/s41598-023-50988-3 (PMC10766980; doi:10.1038/s41598-023-50988-3)
Supplement: Supplementary file 1 — Supplementary Information. [file 41598_2023_50988_MOESM1_ESM.docx]

**Supplementary Information: Infrared-reflective ultrathin-metal-film-based transparent electrode with ultralow optical loss for high efficiency in solar cells**

**George Perrakis^1,*^, Anna C. Tasolamprou^2^, George Kakavelakis^3,4,*^, Konstantinos Petridis^3^, Michael Graetzel^4^, George Kenanakis^1^, Stelios Tzortzakis^1,5,6^, & Maria Kafesaki^1,5^**

^1^Institute of Electronic Structure and Laser (IESL), Foundation for Research and Technology - Hellas (FORTH), 70013 Heraklion, Crete, Greece

^2^Department of Physics, Section of Electronic Physics and Systems, National and Kapodistrian University of Athens, 15784, Athens, Greece

^3^Department of Electronic Engineering, Hellenic Mediterranean University, Romanou 3, Chalepa, GR-73100, Chania, Crete, Greece

^4^Laboratory of Photonics and Interfaces, Institute of Chemical Sciences and Engineering, Ecole Polytechnique Fédérale de Lausanne, 1015, Lausanne, Switzerland

^5^Department of Materials Science and Technology, University of Crete, 70013 Heraklion, Crete, Greece

^6^Texas A&M University at Qatar, 23874 Doha, Qatar

*Corresponding authors: [gperrakis@iesl.forth.gr](mailto:gperrakis@iesl.forth.gr), kakavelakis@hmu.gr

In the Supplementary Information we provide detailed description of the experimental characterization of PSCs w/ Au and MTE. The document includes 6 pages, 3 figure, and 2 sections:

**S1.** Experimental characterization of the absorptivity/emissivity spectra of conventional perovskite solar cells in the visible and infrared wavelength range.

**S2.** Complex refractive indices of the materials used in the simulations.

**S1.** **Experimental characterization of the absorptivity/emissivity spectra of conventional perovskite solar cells in the visible and infrared wavelength range.**

The absorption properties of PSCs in IR (~*λ*_g_–4 μm) have not been examined in literature, despite seriously affecting *T*_c_ and PCE. To ensure validity of our simulations and correctly account for the impact of filtering of IR light, we fabricated two PSC samples (see Fig. S1d) and characterized their solar absorption and thermal emission properties (Fig. S1a–c). We consider two types of state-of-the-art PSCs with typical mesoporous architecture geometry: cell denoted by “w/ Au” is a PSC with Au back contacts for enhanced efficiency, and cell denoted by “w/ MLG” is a PSC with a multilayer Graphene back contact for enhanced stability and low cost. Specifically, cell configuration w/ Au exhibits record efficiencies, even beyond 25% for smaller cells^1,2^, but in expense to the increased cost, and reduced operational stability^3,4^. Cell configurations w/ MLG exhibit lower efficiencies (~20%)^4,5^, mainly due to open-circuit voltage (*V*_OC_) loss as a result of hole trapping due to poor contact (pinholes and gaps) at the hole-transporting layer (HTL)-MLG interface (see Fig. S1d)^5^, but high device operational stability and lower cost due to high-throughput fabrication, e.g., by utilizing printing techniques and low-cost materials^3,4,6^. The rest of the structure is as follows: TEC 8 (FTO – 500 nm)-covered glass substrate (2.2 mm)/compact TiO_2_ (cTiO_2_ – 30 nm)/mesoporous TiO_2_ (mTiO_2_ – 200 nm)/perovskite (FAPbI_3_ – 800 nm)/Octylamonium iodide (OAI – 5 nm)/Spiro-OMeTAD (200 nm)/Au (80 nm) or multi-layer Graphene (10 μm). For both solar cells, low-band gap formamidinium lead iodide perovskite (FAPbI_3_) is the photoactive layer for enhanced efficiency and thermal stability^1,2^. The transparent conducting oxide (commercial FTO: fluorine doped tin oxide), multilayer Graphene (MLG), and gold (Au) layers are the front and rear electrical contacts, respectively, while the transparent soda-lime glass is the encapsulant substrate. OAI is the passivation layer, Spiro-OMeTAD is the hole-transporting layer (HTL), while the compact (cTiO_2_) and mesoporous (mTiO_2_) titanium dioxide layers compose the electron-transporting layer (ETL).

We characterized the structures with an FT-IR spectrometer and compared with the simulated results by employing the transfer matrix method (see also Methods section). To simulate the performance of our structures, we obtained the material parameters used for the active layer from Ref.^7^, of the electrodes from Refs.^8–13^ and of the other layers from Refs.^8,9,14^, see also Fig. S2 and S3. Examined PSCs’ experimental and simulated solar absorption and thermal emissivity spectra are shown in Fig. S1.


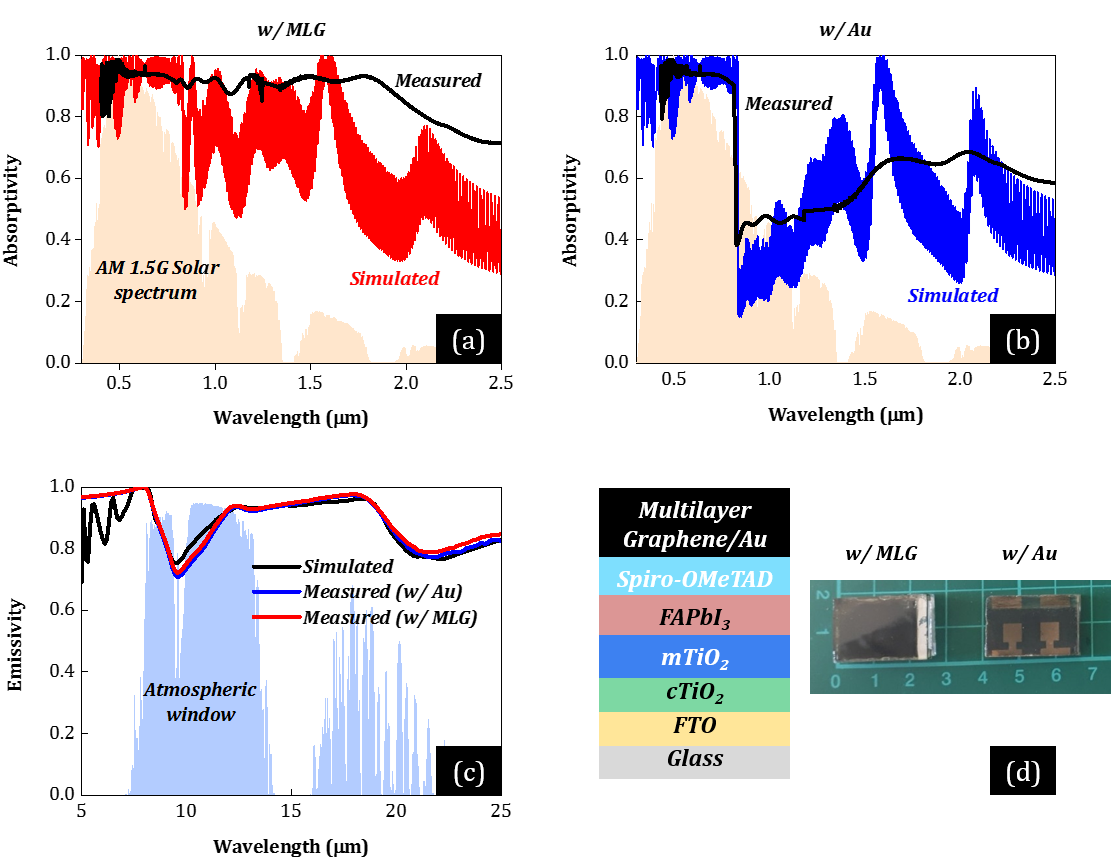


**Figure S1.** (**a, b**) Experimentally measured (black) and simulated (red and blue) solar absorption spectra of the two PSC samples w/ MLG (red) and Au (blue), with the normalized AM1.5G solar spectrum plotted for reference (orange shaded area). Note that the PSC w/ Au absorbs much less light of one-micron wavelength than the PSC w/ MLG due to reflection from the device. (**c**) Simulated (black) and experimentally measured (red and blue) thermal emissivity spectra of the two PSC samples w/ MLG (red) and Au (blue), with realistic atmosphere transmittance plotted for reference (blue shaded area). (**d**) Geometry of the PSCs investigated here (left). The role of the different layers of the solar cells is discussed in the main text. Photo of the back side of the two measured solar cells (right). Cell “w/ MLG”: a perovskite solar cell with a multilayer Graphene back contact. Cell “w/ Au”: a perovskite solar cell with Au back contacts.

First at *λ*<*λ*_g_, the experimental (and simulated) absorptivity in both PSC cases is high and comparable (i.e., no matter Au or MLG), which is attributed to FAPbI_3_ of enhanced transport properties that allow for large perovskite layer thicknesses (<800 nm – see Supplementary Information) and therefore efficient light absorption without lowering the EQE^1,2^. At *λ*>*λ*_g_, the experimental absorptivity (black) in both PSC cases validate the high NIR and SWIR absorption in state-of-the-art PSCs as predicted by the simulations (red/blue). Deviations in the absorptivity spectra, i.e., less pronounced absorptivity peaks (originating from interference in the thin film stack) and a systematic higher absorptivity of the characterized samples compared to simulations could also be attributed to the samples’ roughness, whereas in the simulations planar interfaces were assumed. For instance, the thicker MLG (~10-μm-thick compared to ~80-nm-thick Au) in general results to higher surface roughness (also one of the reasons for the lower *V*_OC_ output in PSCs w/ MLG), which could be also the reason for the larger difference in absorptivity between simulation and experiment in case of “w/ MLG” (Fig. 2a) compared to “w/ Au” (Fig. 2b), especially in NIR and SWIR where perovskite does not absorb. Moreover, we note that a better agreement is expected if measured material properties (permittivity or refractive index of each material) are utilized^15^. The absorptivity decrease at *λ*>1.9 μm in all cases (measured and simulated) is attributed to the epsilon-near-zero (ENZ) response of conductive FTO in that regime^8^.

In the solar wavelength region (~0.3−4 μm, see Fig. S1a,b), both PSCs show strong light absorption, especially at ~0.3−*λ*_g_ μm, as expected. In IR (~*λ*_g_−4 μm), both PSCs show high absorption, despite IR photons’ lower energy than active material’s band gap energy. For the cell w/ Au, such strong IR absorption mainly originates from the FTO front contact^16^, representing a heat source. The sub-band gap absorption further increases due to interference in the thin-film stack. According to our calculations (i.e., from the simulated absorption in other layers than FAPbI_3_ in 0.3–4 μm and the AM1.5G sun spectrum), the parasitic absorption in the cell w/ Au equals 200 W/m^2^. This absorption does not contribute to photocurrent and acts as a heat source, seriously affecting solar cells’ PCE and reliability. The cell w/ MLG shows an even stronger absorption in IR (see Fig. S1a), expected to lead to excess heat generation and even higher operating temperatures in realistic outdoor conditions. The reason is that MLG strongly absorbs in NIR compared to Au, which acts as a reflector. As a result, in the cell w/ MLG, parasitic absorption equals an even higher value of 310.6 W/m^2^ (i.e., 110.6 W/m^2^ more than the cell w/ Au). We note that PSCs’ heat output is expected to be slightly higher than 200 and 310.6 W/m^2^ due to higher experimental absorptivity than the simulations (see Fig. S1a,b).

In MIR at ~4−25 μm (see Fig. S1c), PSCs’ front surface thermal emissivity comes almost exclusively from the millimeter-thick glass substrate. Additionally, PSC devices’ emissivity is almost identical to all solar cell technologies (see Fig. S1c) since glass is the most common/reliable encapsulant/substrate. These bulk layers have relatively strong thermal emissivity due to their phonon-polariton resonance modes at MIR^17,18^, and hence have a cooling effect. However, the thermal emissivity of these layers is typically not optimized, especially within the atmospheric transparency window, which lowers their cooling power (see Fig. S1c).

In summary, the results of Fig. S1 indicate that most promising PSCs inevitably (to be efficient, cost-effective, and functional) have (i) strong parasitic absorption in the solar wavelengths and (ii) sub-optimal emissivity in the thermal wavelengths (~4−25 μm). As a result, even thin-film PSCs technology (~1 μm) suffers intense thermal loads on a sunny day^19^, comparable to bulkier silicon-based counterparts (~250 μm thickness)^20^, seriously affecting their PCE and reliability^21,22^.

One way to reduce parasitic absorption in the cell w/ Au is to mitigate the *T*-*R*_sh_ trade-off effect by utilizing an appropriate front TCO layer with higher transparency at a given conductivity compared to FTO. Common TCOs, though, such as optically superior indium-doped tin oxide (ITO) or aluminum-doped zinc oxide (AZO, ZnO:Al), limit PSC performance and application due to conductivity loss at high annealing temperatures required for the compact (cTiO_2_) or mesoporous TiO_2_ (mTiO_2_) layers and increase the cost. Substituting cTiO_2_ and mTiO_2_ with other alternatives, such as PTAA or SnO_2_, seems promising for utilizing various TCOs such as ITO^23^, reducing PSCs’ solar heating due to lower parasitic absorption in the front contact. However, like the examined cell w/ Au (Fig. S1b), such cells exhibit unsatisfactory operational stability, limited large-scale application, and high cost, mainly due to the metal back contact^3^, impeding commercial exploitation. Substituting the metal back contact of such cells with MLG to enhance their operational stability and reduce the cost leads to excess heat since MLG strongly absorbs in NIR. Moreover, the sheet resistance of conventional electrodes and TCOs (~8–15 Ω/sq) is still high when upscaling from a single cell to a module^24^. This results to lower module solar-to-electrical PCEs (due to less efficient carrier transport) and further increases heat dissipation, thus indicating that structural changes or other TCO candidates are required. These results indicate that an ultrathin-metal-film-based multilayer transparent electrode (MTE) as discussed in the main text, should highly impact PSCs PCE and reliability.

**S2. Complex refractive indices of the materials used in the simulations.**

To simulate the performance of our structures, we obtained the material parameters used for the active layer from Ref.^7^, of the electrodes from Refs.^8–13^ and of the other layers from Refs.^8,9,14^, shown in Fig. S2 and S3.


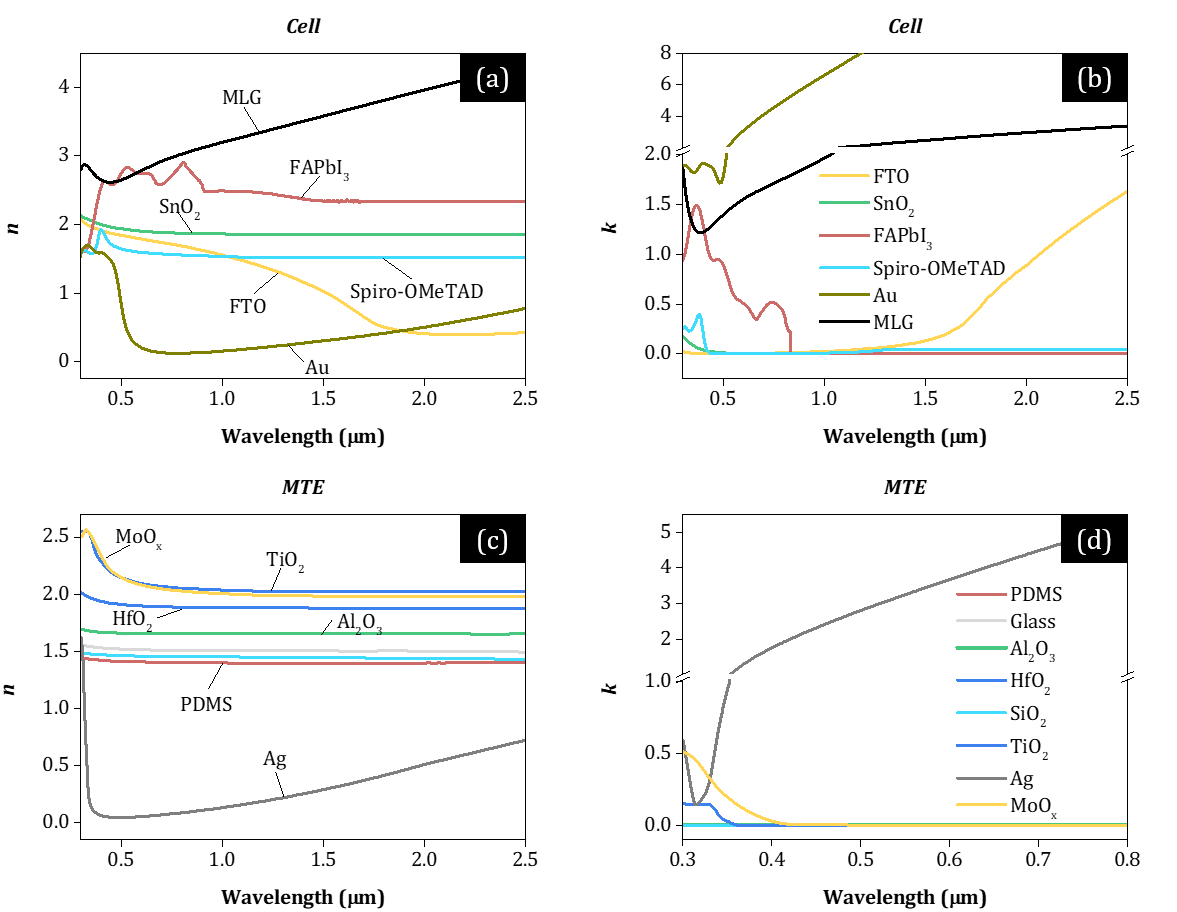


**Figure S2.** Refractive index (*n*) and extinction coefficient (*k*) of the materials used for simulating (**a**, **b**) the PSC cells’ and (**c**, **d**) MTEs’ optical response at solar wavelengths.


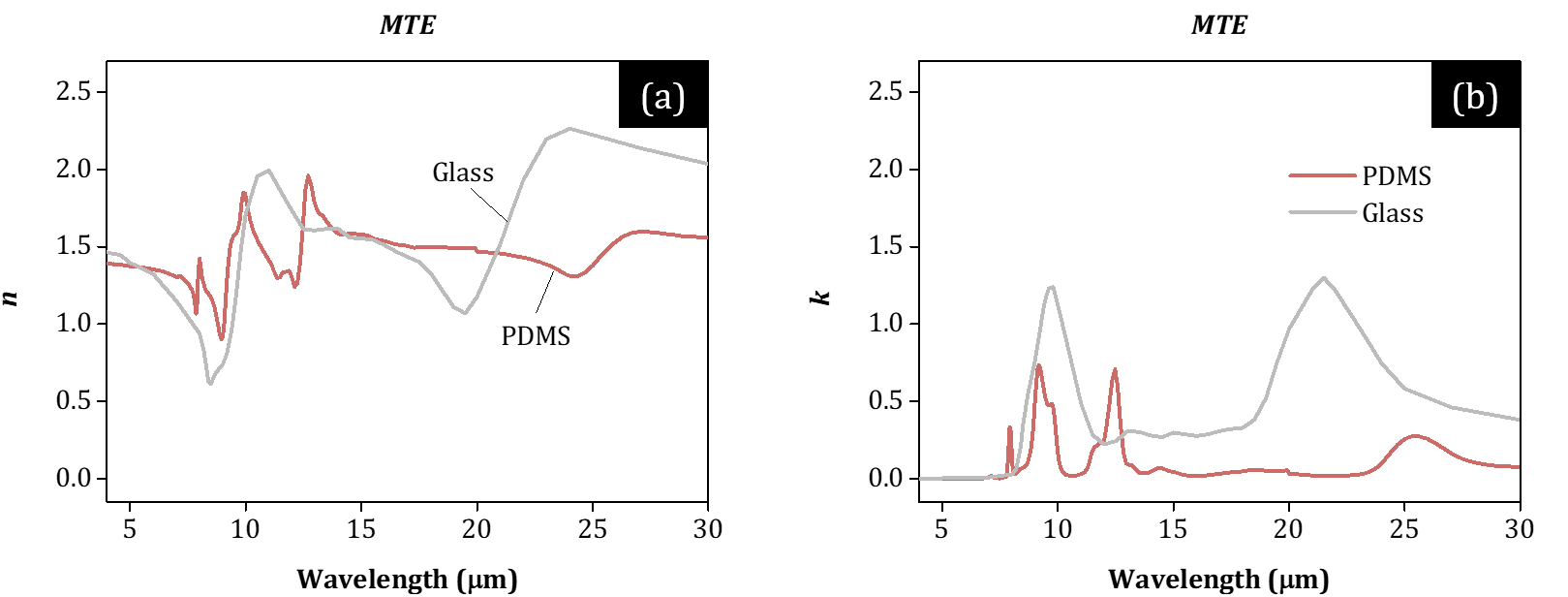


**Figure S3.** Refractive index (*n*) and extinction coefficient (*k*) of the materials used for simulating (**a**, **b**) the PSC cells’ and MTEs’ optical response at thermal wavelengths.

**References**

1. Jeong, J. *et al.* Pseudo-halide anion engineering for α-FAPbI3 perovskite solar cells. *Nat. 2021 5927854* **592**, 381–385 (2021).

2. Min, H. *et al.* Perovskite solar cells with atomically coherent interlayers on SnO2 electrodes. *Nat. 2021 5987881* **598**, 444–450 (2021).

3. Fagiolari, L. & Bella, F. Carbon-based materials for stable, cheaper and large-scale processable perovskite solar cells. *Energy Environ. Sci.* **12**, 3437–3472 (2019).

4. Yu, Y., Hoang, M. T., Yang, Y. & Wang, H. Critical assessment of carbon pastes for carbon electrode-based perovskite solar cells. *Carbon N. Y.* **205**, 270–293 (2023).

5. Zhang, C. *et al.* Ti1–graphene single-atom material for improved energy level alignment in perovskite solar cells. *Nat. Energy 2021 612* **6**, 1154–1163 (2021).

6. Ku, Z., Rong, Y., Xu, M., Liu, T. & Han, H. Full Printable Processed Mesoscopic CH3NH3PbI3/TiO2 Heterojunction Solar Cells with Carbon Counter Electrode. *Sci. Reports 2013 31* **3**, 1–5 (2013).

7. Subedi, B. *et al.* Formamidinium + cesium lead triiodide perovskites: Discrepancies between thin film optical absorption and solar cell efficiency. *Sol. Energy Mater. Sol. Cells* **188**, 228–233 (2018).

8. Jiang, Y. *et al.* Optical analysis of perovskite/silicon tandem solar cells. *J. Mater. Chem. C* **4**, 5679–5689 (2016).

9. Perrakis, G. *et al.* Submicron Organic-Inorganic Hybrid Radiative Cooling Coatings for Stable, Ultrathin, and Lightweight Solar Cells. *ACS Photonics* **9**, 1327–1337 (2022).

10. Pisoni, S. *et al.* Tailored lead iodide growth for efficient flexible perovskite solar cells and thin-film tandem devices. *NPG Asia Mater. 2018 1011* **10**, 1076–1085 (2018).

11. Djurišić, A. B. & Li, E. H. Optical properties of graphite. *J. Appl. Phys.* **85**, 7404–7410 (1999).

12. Jiang, Y., Pillai, S. & Green, M. A. Realistic Silver Optical Constants for Plasmonics. *Sci. Reports 2016 61* **6**, 1–7 (2016).

13. Nash, D. J. & Sambles, J. R. Surface plasmon-polariton study of the optical dielectric function of silver. *J. Mod. Opt.* **43**, 81–91 (1996).

14. Raoult, E. *et al.* Optical Characterizations and Modelling of Semitransparent Perovskite Solar Cells for Tandem Applications. *36th Eur. Photovolt. Sol. Energy Conf. Exhib.* 757–763 (2019). doi:10.4229/EUPVSEC20192019-3BV.2.53

15. Jošt, M. *et al.* Textured interfaces in monolithic perovskite/silicon tandem solar cells: advanced light management for improved efficiency and energy yield. *Energy Environ. Sci.* **11**, 3511–3523 (2018).

16. Ma, C. *et al.* Light management in highly-textured perovskite solar cells: From full-device ellipsometry characterization to optical modelling for quantum efficiency optimization. *Sol. Energy Mater. Sol. Cells* **230**, 111144 (2021).

17. Zhu, L., Raman, A. P. & Fan, S. Radiative cooling of solar absorbers using a visibly transparent photonic crystal thermal blackbody. *Proc. Natl. Acad. Sci. U. S. A.* **112**, 12282–7 (2015).

18. Li, W., Shi, Y., Chen, K., Zhu, L. & Fan, S. A Comprehensive Photonic Approach for Solar Cell Cooling. *ACS Photonics* **4**, 774–782 (2017).

19. Pescetelli, S. *et al.* Integration of two-dimensional materials-based perovskite solar panels into a stand-alone solar farm. *Nat. Energy 2022* 1–11 (2022). doi:10.1038/s41560-022-01035-4

20. Xu, L. *et al.* Heat generation and mitigation in silicon solar cells and modules. *Joule* **5**, 631–645 (2021).

21. Chen, B. *et al.* Synergistic Effect of Elevated Device Temperature and Excess Charge Carriers on the Rapid Light-Induced Degradation of Perovskite Solar Cells. *Adv. Mater.* **31**, 1902413 (2019).

22. Lin, Y. *et al.* Excess charge-carrier induced instability of hybrid perovskites. *Nat. Commun. 2018 91* **9**, 1–9 (2018).

23. Li, Z. *et al.* Organometallic-functionalized interfaces for highly efficient inverted perovskite solar cells. *Science (80-. ).* **376**, (2022).

24. Lee, S. H., Kim, G., Lim, J. W., Lee, K. S. & Kang, M. G. High-performance ZnO:Ga/Ag/ZnO:Ga multilayered transparent electrodes targeting large-scale perovskite solar cells. *Sol. Energy Mater. Sol. Cells* **186**, 378–384 (2018).
